# NMR Spectroscopy for Studying the Selective Etching of Ti_3_AlC_2_ to Ti_3_C_2_T*_x_* MXene Using Hexafluorosilicic Acid

**DOI:** 10.1002/smtd.202501640

**Published:** 2026-01-04

**Authors:** Henry J. Hamann, Anupma Thakur, Nithin Chandran B S, Krutarth Kamath, Babak Anasori, P. Veeraraghavan Ramachandran

**Affiliations:** ^1^ Department of Chemistry Purdue University West Lafayette Indiana United States; ^2^ School of Materials Engineering Purdue University West Lafayette Indiana United States; ^3^ School of Mechanical Engineering Purdue University West Lafayette Indiana United States

**Keywords:** etching, hexafluorosilicic acid, MXene, NMR spectroscopy, 2D materials

## Abstract

The stringent safety protocols required for hydrofluoric acid (HF) based MAX phase etching and the reliance on material characterization tools such as XRD, SEM, EDS and XPS to study etching make the MXene research challenging. Here, we have employed ^27^Al NMR spectroscopy for the rapid detection of selective etching, directly from the etching supernatant, of soluble aluminum species generated during the MAX phase etching reaction. This technique was applied to the development of a new etching protocol for Ti_3_AlC_2_ MAX phase using the less hazardous hexafluorosilicic acid. The etching process was studied using a combination of ^27^Al and ^19^F NMR spectroscopies where it was demonstrated to be free of HF or free fluoride in quantities detectable by ^19^F NMR, and that the primary etching byproduct is H_3_AlF_6_. ^19^F NMR spectroscopy was additionally proven to be a viable technique to quantify the extent of etching using trifluoroacetic acid as an internal standard.

## Graphical abstract

^27^Al NMR spectroscopy is introduced as a method to detect the etching of Ti_3_AlC_2_ to Ti_3_C_2_T*_x_* MXene. Soluble aluminum species produced during etching were identified by sampling the etching supernatant during the reaction. Using a combination of ^27^Al and ^19^F NMR spectroscopies, a new acid etchant, hexafluorosilicic acid, was shown to be free of HF or free fluoride in quantities detectable by ^19^F NMR before and during the etching reaction, with H_3_AlF_6_ as the primary etching byproduct.

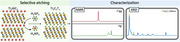

## 1 Introduction

Since their discovery in 2011 [1], MXenes, 2D transition metal carbides, nitrides, or carbonitrides [2], have found use in wide‐ranging applications, including energy storage [3, 4, 5, 6, 7, 8, 9, 10], lubricants [11, 12], electromagnetic interference shielding [13, 14], water purification [15, 16, 17], and as catalysts [18, 19]. The flagship titanium carbide (Ti_3_C_2_T*_x_*) MXene [20] and a majority of other MXenes reported have been prepared by selective etching of layered carbides or nitrides, so‐called MAX phases, using aqueous hydrofluoric acid (HF), fluoride‐based salts, and HF mixed with other acids.

A goal of materials chemists/engineers has been the development of HF‐free etching protocols owing to its extreme toxicity [21]. To this end, alternative acids (H_3_PO_4_ [22], CF_3_COOH [23]), alternative etchants (NH_4_HF_2_ [24], I_2_ [25], (Br_2_, I_2_, ICl, IBr) [26], LiPF_6_ [27]), hydrothermal alkali reactions [28], in situ formation of HF using fluoride salts (LiF [29], NaF [30], KF [31], NH_4_F [32], NaBF_4_ [33]) with HCl, the use of ionic liquids [34], and molten salt (ZnCl_2_ [35], CdBr_2_ [36], LiF/KF [37], NH_4_HF_2_ [38]) synthesis have been explored. However, most of these alternative protocols are limited in scope [22] and require high temperatures (>200 °C) [28, 35, 36] or pose technical challenges [35, 36, 37], limiting their general adoption for the scalable synthesis of MXenes.

Development of new etching protocols is reliant on material characterizations, such as X‐ray diffraction (XRD) patterns, scanning electron microscopy (SEM) imaging with energy dispersive X‐ray spectroscopy (EDS) [39]. Visual indicators, darkened color or “clay‐like” appearance of etched materials, and presence of the Tyndall effect, can be quantitatively unreliable predictors of successful etching. Drawing on our prior experience using solution phase ^27^Al nuclear magnetic resonance (NMR) spectroscopic analysis [40], we hypothesized that this technique could be applied to MAX phase etching. This was confirmed by analysis of the supernatant of etched Ti_3_AlC_2_ MAX using HF and HCl as mixed acid etchants (Figures S1 and S2) [41].

## 2 Results and Discussion

Initial attempts at etching the Ti_3_AlC_2_ MAX phase using carboxylic acids showed peaks in the ^27^Al NMR spectra of the reaction supernatants, but the small quantity of aluminum etched using these acids yielded no discernible peaks in the XRD diffraction pattern corresponding to MXene (Tables S1 and S2 and Figures S3–S15). This led us to reevaluate our approach. We transitioned to using hexafluorosilicic acid (H_2_SiF_6_), which is a bulk chemical byproduct of phosphoric acid and superphosphate fertilizer manufacturing [42] consumed primarily in the production of synthetic cryolite and aluminum trifluoride (AlF_3_). H_2_SiF_6_ finds additional application in the production of fluorosilicates [42] and water fluoridation [43]. The safety data sheet of H_2_SiF_6_ indicates that it is less hazardous (oral, dermal, and inhalation) compared with aqueous HF [44, 45]

**FIGURE 1:**
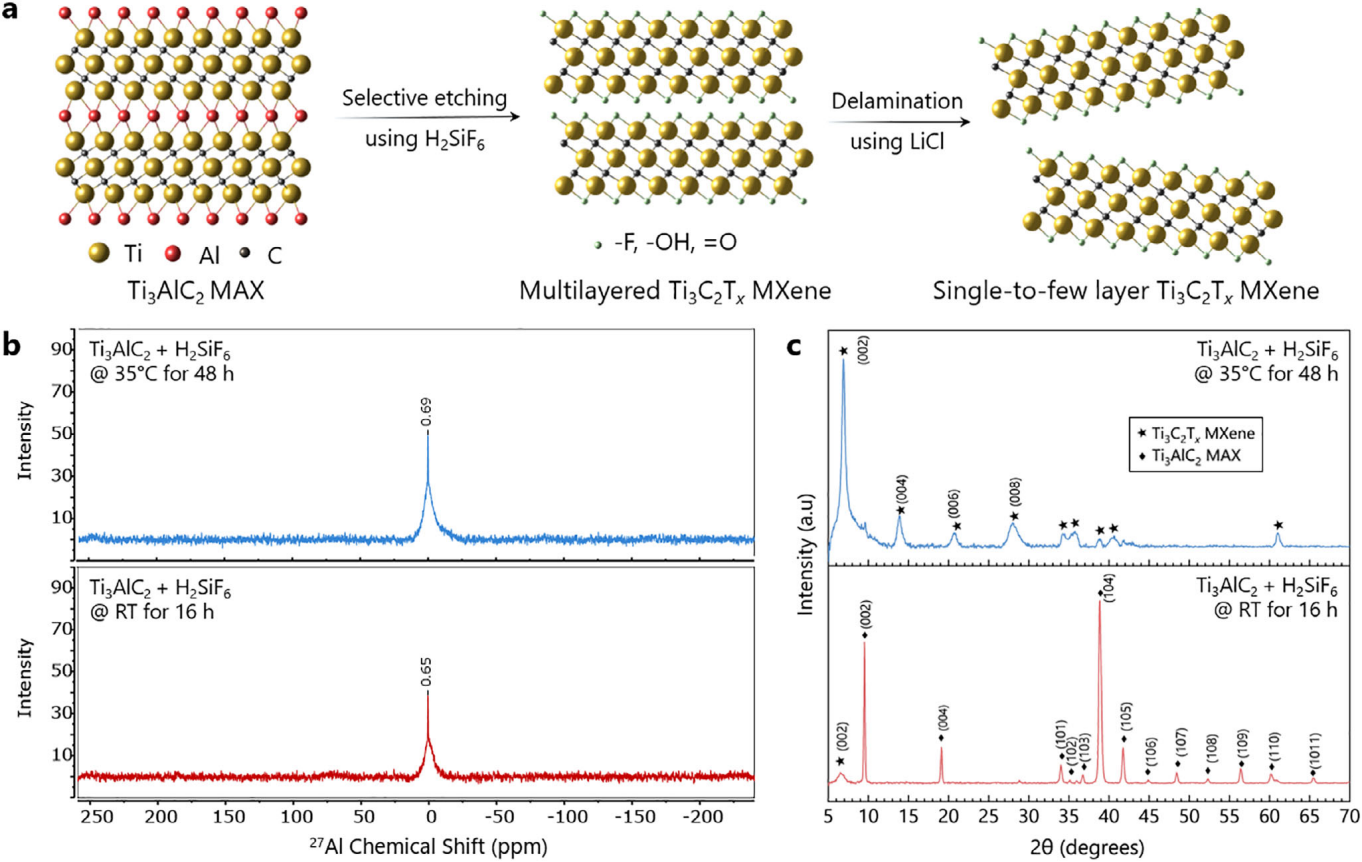
(a) Schematic representation of selective etching of Ti_3_AlC_2_ MAX phase to multilayered Ti_3_C_2_T*_x_* MXene using H_2_SiF_6_ followed by delamination to single‐to‐few‐layered Ti_3_C_2_T*_x_* MXene. (b) ^27^Al NMR spectra from the supernatant of initial (bottom) and optimized (top) H_2_SiF_6_ etching reactions. (c) XRD patterns from initial partial etching (bottom) and optimized completely etched multilayered Ti_3_C_2_T*_x_* MXene (top) using H_2_SiF_6_.

Here, we report our successful application of ^27^Al and ^19^F NMR spectroscopy as a characterization tool and of H_2_SiF_6_ for Ti_3_AlC_2_ MAX phase selective etching to synthesize multilayered Ti_3_C_2_T*_x_* MXene, and upon delamination using LiCl, single‐ to few‐layer Ti_3_C_2_T*_x_* MXene flakes (Figure 1a). ^27^Al NMR spectra obtained from the supernatant of H_2_SiF_6_ etched Ti_3_AlC_2_ MAX phase under initial (16 h, room temperature (RT)) (Figure 1b, bottom) and optimized (48 h, 35 °C) (Figure 1b, top) conditions each show prominent peaks at chemical shift values of δ 0.65–0.69 ppm. The capability of ^27^Al NMR spectroscopy as a highly sensitive tool for the detection of Ti_3_AlC_2_ MAX phase etching is evident when comparing the ^27^Al NMR spectra in Figure 1b with the corresponding XRD pattern in Figure 1c.

The XRD pattern obtained for the Ti_3_AlC_2_ MAX phase, prepared using excess metals (Ti and Al) [41], etched under optimized conditions (48 h at 35 °C) (Figure 1c, top) shows nearly complete disappearance of the characteristic (002) peak at 9.51° for the Ti_3_AlC_2_ MAX phase and the appearance of the (002) Ti_3_C_2_T*_x_* MXene peak at a lower 2θ (6.96°). The XRD pattern obtained for the Ti_3_AlC_2_ MAX phase etched under the initial unoptimized conditions shows (002) peaks for both Ti_3_AlC_2_ MAX phase and Ti_3_C_2_T*_x_* MXene. The modest partial etching observed using XRD was detected as soluble aluminum species in the corresponding ^27^Al NMR spectrum.

**FIGURE 2:**
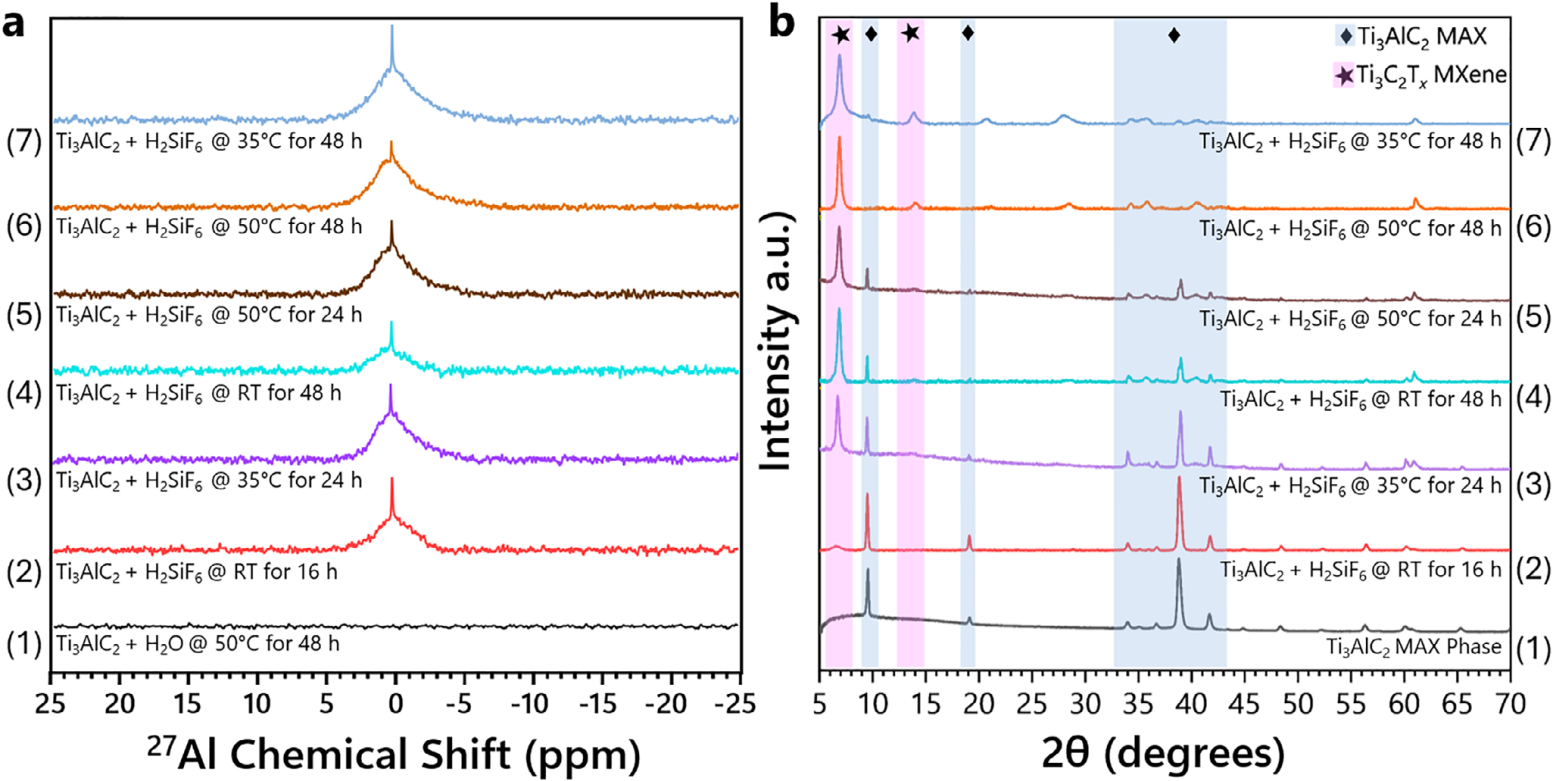
Spectroscopic analysis from optimization of Ti_3_AlC_2_ etching using H_2_SiF_6_. Etching reactions used 10 mL of H_2_SiF_6_ and 0.25 g of Ti_3_AlC_2_. (a) ^27^Al NMR spectra obtained from etching supernatant. (b) X‐ray diffraction patterns obtained from the isolated reaction material.

Baseline controls were obtained by acquiring the XRD pattern of Ti_3_AlC_2_ MAX as well as the ^27^Al NMR spectrum of the supernatant of its aqueous suspension etched at 50 °C for 48 h. The XRD and ^27^Al NMR spectra (Figure 2 row 1) reveal the expected reflections for Ti_3_AlC_2_ MAX and the absence of aluminum in the supernatant solution. The ^27^Al NMR spectrum (Figure 2a row 2) of the initial Ti_3_AlC_2_ etching reaction in aqueous H_2_SiF_6_ at RT for 16 h shows two overlapping peaks centered near δ 0.65–0.69 ppm. Following repeated centrifugation with deionized (DI) water, the collected material was characterized using XRD (Figure 2b row 2), which confirmed the evidence of Ti_3_C_2_T*_x_* MXene, as indicated by the broad (002) reflection observed at a 2θ of 6.96°.

Gradual modification of the etching conditions was performed by adjusting the etching time and temperature (Table S3 and Figure S16). The ^27^Al NMR spectra obtained for the supernatant of each reaction (Figure 2a) confirmed the presence of etched aluminum. The peaks in each spectrum are near the same chemical shift (∼δ 0.65–0.69 ppm) and have approximately the same intensity. The XRD analysis (Figure 2b) revealed that with increasing reaction temperature and time, the (002) peak corresponding to the MAX phase at 2θ ≈ 9.51° gradually diminishes, while a new low‐angle (002) peak emerges, indicative of Ti_3_C_2_T*_x_* MXene.

The modest etching observed for the initial 16 h, RT condition was increased by elevating the reaction temperature to 35 °C for 24 h or etching at RT for 48 h (Figure 2, rows 3,4). For 24 and 48 h reactions at ∼50 °C (Figure 2 rows 5,6), etching was complete or nearly complete, however, a white solid was formed at the surface and top cover of the reaction bottle. SEM, EDS, Raman, and XRD analysis showed it to be amorphous SiO_2_ and sub‐stoichiometric SiO*_x_* (Figures S17–S20) [46, 47, 48]. The optimization study ultimately revealed that 0.25 g of Ti_3_AlC_2_ MAX is completely etched in 10 mL of 35 wt.% H_2_SiF_6_ after 48 h at 35 °C, while minimizing byproduct formation (Figure 2 row 7).

Experiments varying the reaction concentration were performed using 0.5 g Ti_3_AlC_2_ MAX, or 20 mL of 35 wt.% H_2_SiF_6_, and are shown in Figure S16. In addition to H_2_SiF_6_, the related H_2_TiF_6_ and H_2_ZrF_6_ were also demonstrated to be viable alternative etchants under conditions similar to those of the optimized protocol (Table S4 and Figures S21–S26).

The optimized etching protocol was subsequently performed on different scales of MXene synthesis using 1, 1.25, and 4 g of Ti_3_AlC_2_ MAX. The wet‐etched multilayered Ti_3_C_2_T*_x_* MXene powder from each batch was delaminated using lithium chloride (LiCl) to separate single‐to‐few‐layered flakes and multilayered Ti_3_C_2_T*_x_* MXene clay sediment. The single‐to‐few‐layered Ti_3_C_2_T*_x_* MXene suspended in the supernatant was separated, and synthesis yields were measured to be consistent ∼ 25 ± 3% across all the batches.

**FIGURE 3:**
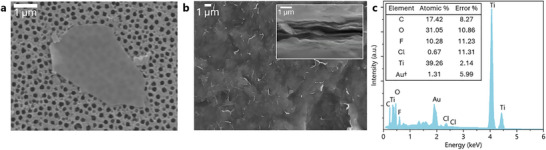
Characterization of Ti_3_C_2_T*_x_* MXene synthesized using the optimized H_2_SiF_6_ etching conditions. (a) SEM image of single‐to‐few layered Ti_3_C_2_T*_x_* MXene flake obtained after delamination. (b) SEM image of the surface of the free‐standing Ti_3_C_2_T*_x_* MXene film (inset: cross‐sectional view of the prepared Ti_3_C_2_T*_x_* MXene film). (c) EDS spectrum obtained from the surface of free‐standing Ti_3_C_2_T*_x_* MXene and the measured elemental composition. ^†^Gold (Au) is present as a result of sputtering during sample preparation.

SEM analysis of the single‐to‐few‐layered Ti_3_C_2_T*_x_* MXene suspension (Figure 3a) showed electron‐transparent flakes with no visible holes and defects within the 2D flakes. Additional images from the SEM analysis of the single‐to‐few‐layered Ti_3_C_2_T*_x_* MXene suspension, showing flakes of equal quality, are seen in Figure S27a–c. Several images (Figure S28a,b) show nanoparticles present on the flask surface. As Ti_3_C_2_T*_x_* MXene oxidation begins preferentially on a defective edge, not on the surface, these nanoparticles are most likely residual from the synthesis and can be removed by additional washing steps. Further investigation of the MXene quality was performed from the vacuum‐assisted preparation of a free‐standing Ti_3_C_2_T*_x_* MXene film from the colloidal suspension of the single‐to‐few‐layered MXene (Figure 3b). The electrical conductivity of the prepared free‐standing Ti_3_C_2_T*_x_* MXene was measured to be ∼ 7000 ± 250 S/cm. EDS analysis of the prepared Ti_3_C_2_T*_x_* MXene film (Figure 3c) detected titanium and carbon, as well as oxygen and fluorine, suggesting a mixture of fluorine and oxygen‐containing surface termination groups.

A Raman spectrum of synthesized Ti_3_C_2_T*_x_* MXene (Figure S29) was collected using a 785 nm laser with a grating size of 1200 gr/mm and power 0.1%. The observed Raman spectrum can be segmented into three distinct regions: the flake region, which corresponds to a collective vibration of carbon, two titanium layers, and surface groups; the T*_x_* region, which represents the vibrations of surface groups (─O, ─OH, ─F, ─Cl), and the carbon region, which encompasses both in‐plane and out‐of‐plane vibrations of carbon atoms. The minimal presence of free carbon (D and G peak) indicates that the synthesized Ti_3_C_2_T*_x_* MXene films are stable and not subjected to hydrolysis or oxidation [49, 50].

A comparison of the H_2_SiF_6_‐based etching of Ti_3_AlC_2_ MAX phase with the widely adopted HF‐based protocol reveals a slight decrease in the synthesis yield of the single‐to‐few‐layered Ti_3_C_2_T*_x_* MXene of ∼25 ± 3%, compared with ≈38% yield for the HF‐based protocol [41]. However, several factors may offset this discrepancy. The uniquely dangerous nature of HF requires specialized antidotes and personal protective equipment, as well as strict standard operating procedures, as even a minor mishandling can be life‐threatening [45]. H_2_SiF_6_, while still a highly corrosive acid, does not impart systemic toxicity from trace exposure, like HF, and can generally be managed using standard corrosive‐acid safety measures [44]. The margin of safety between HF and H_2_SiF_6_ in turn affects the overall cost of the materials, with HF being generally more costly due to transport and handling restrictions and necessary engineering controls. A final factor for consideration is the potential for further improvement to the H_2_SiF_6_‐based etching, which has not been studied to the same extent as the HF‐based protocol.

**FIGURE 4:**
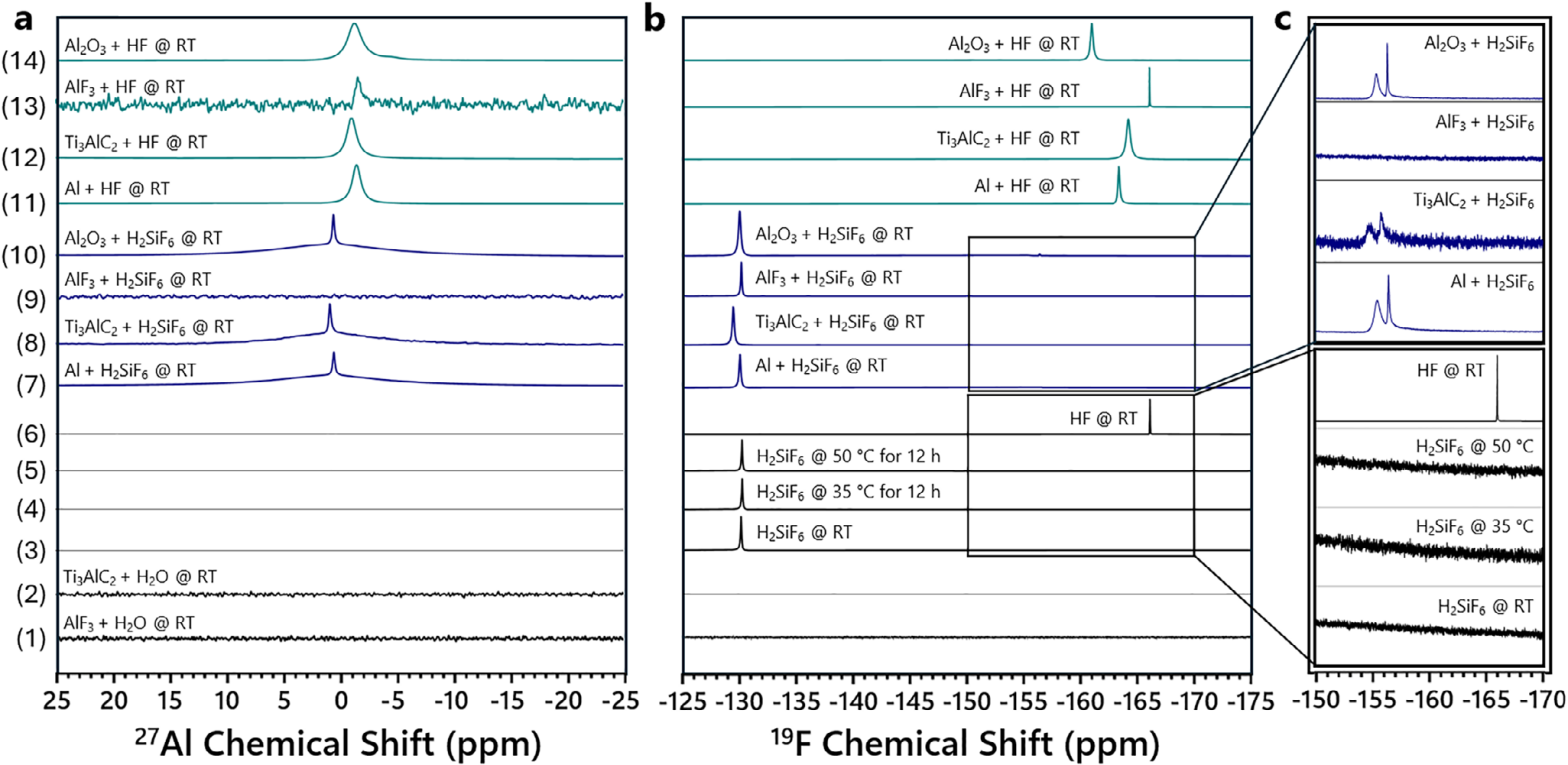
^27^Al and ^19^F NMR spectroscopy study of the H_2_SiF_6_ etching protocol. (a) ^27^Al NMR spectra and (b) ^19^F NMR spectra obtained from the aluminum and fluorine controls and the reactions of Al metal, Ti_3_AlC_2_ MAX, AlF_3_, and Al_2_O_3_ with both H_2_SiF_6_ and HF. (c) Highlighted portions of the ^19^F NMR spectra demonstrating the absence of HF from the H_2_SiF_6_ solution and the reactions of Al metal, Ti_3_AlC_2_ MAX, AlF_3_, and Al_2_O_3_ with H_2_SiF_6_.

Insight into the selective etching process was obtained via ^27^Al and ^19^F NMR spectroscopic analysis of the supernatant from reactions of Al metal, Ti_3_AlC_2_ MAX, AlF_3_, and Al_2_O_3_ with both H_2_SiF_6_ and HF (Figure 4). Baseline ^27^Al NMR controls, AlF_3_ or Ti_3_AlC_2_ MAX in DI water (Figure 4a rows 1,2), each showed no detected Al species. The ^19^F NMR spectrum of AlF_3_ was additionally absent of any peaks. ^19^F NMR controls, aqueous H_2_SiF_6_ or HF, showed strong peaks at δ −130.09 and δ −166.02 ppm, respectively (Figure 4b rows 3,6). Critically, no HF was detected in the ^19^F NMR spectra obtained for H_2_SiF_6_ at RT, 35°C, and 50°C (Figure 4b rows 3–5). Figure 4c highlights this absence in the relevant spectral area; the ^19^F NMR spectrum of HF is shown for comparison.

The reactions of Al metal, Ti_3_AlC_2_ MAX, and Al_2_O_3_ with H_2_SiF_6_ (Figure 4a rows 7, 8, and 10) each displayed the same two overlapping peaks centered near δ 0.55 to 0.92 ppm in the ^27^Al NMR spectra. Aluminum was again absent from the spectrum obtained for the reaction of AlF_3_ (Figure 4a row 9). The corresponding ^19^F NMR spectra each exhibited a peak for H_2_SiF_6_ at ∼δ −130 ppm (Figure 4b rows 7–10). The ^19^F NMR spectrum for the reaction of AlF_3_ with H_2_SiF_6_ (Figure 4b row 9) contained no additional peaks, while the spectra for the reactions of Al metal, Ti_3_AlC_2_ MAX, and Al_2_O_3_ (Figure 4b rows 7, 8, and 10) each showed two new peaks at ∼δ −155 and −156 ppm. Figure 4c displays the scaled and zoomed‐in spectral area where these peaks are observed; the absence of HF is also evident in this region. These two peaks correspond to the byproducts of the developed etching protocol, likely hydrates of H_3_AlF_6_, which has earlier been proposed as the byproduct of HF etching protocols [51].

The ^27^Al NMR spectra obtained for the reactions of Al metal, Ti_3_AlC_2_ MAX, and Al_2_O_3_ with HF were similar to those for H_2_SiF_6_ (Figure 4a rows 11–14). A single large peak centered near δ −1.59 to δ −1.07 ppm was observed. The spectrum obtained for the reaction of AlF_3_ showed a very minor peak near the same chemical shift value corresponding to a small quantity of solubilized AlF_3_.

The corresponding ^19^F NMR spectra for each reaction (Figure 4b rows 11, 12, and 14) exhibited a peak centered between δ −164.17 and δ −160.98 ppm. It is proposed that these peaks correspond to H_3_AlF_6_, with the minor changes in chemical shift due to the variation in solution concentration (Figure S31). The ^19^F NMR spectrum for the reaction of AlF_3_ with HF displayed only the HF peak (Figure 4b row 13). Any soluble AlF_3_, as seen in the ^27^Al NMR, is below the limit of detection relative to the strong signal from HF.

Substantial conclusions drawn from this NMR study are that (1) HF or free fluoride, if present, are in quantities below the detection limit of ^19^F NMR in the reactions relevant to the H_2_SiF_6_ etching protocol, although they may still play a role mechanistically. (2) AlF_3_, if present as a byproduct of H_2_SiF_6_ or HF etching, remains undissolved as a solid material [52]. (3) The likely byproduct of either H_2_SiF_6_ or HF etching processes is H_3_AlF_6_. This prospect was examined by dissolving aluminum metal in aqueous H_2_SiF_6_, followed by precipitation of the acidic components using sodium carbonate. Analysis of the XRD pattern of the solid obtained after neutralization confirmed the presence of Na_3_AlF_6_ (Figures S32–S38 and Tables S5–S7), along with Na_2_SiF_6_ as the main constituent and minor quantities of NaF produced during neutralization.

**FIGURE 5:**
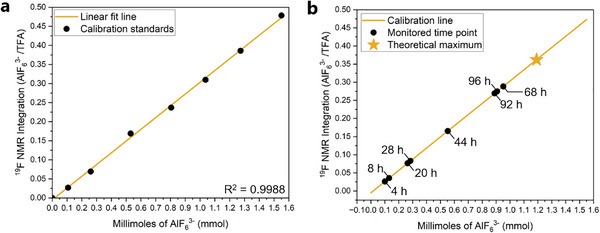
^19^F NMR spectroscopy applied to the quantitative determination of the extent of etching of Ti_3_AlC_2_ MAX phase by H_2_SiF_6_. (a) Calibration curve prepared using trifluoroacetic acid as an internal standard and measured quantities of Na_3_AlF_6_ as the analyte. The known quantities of Na_3_AlF_6_ are plotted against the ratio of AlF_6_^3−^ to trifluoroacetic acids peaks observed in the ^19^F NMR spectra. (b) ^19^F NMR monitored etching reaction of Ti_3_AlC_2_ MAX phase using H_2_SiF_6_. The ratio of AlF_6_^3−^ to trifluoroacetic acids peaks observed in the ^19^F NMR spectra are plotted along the calibration linear regression line to determine the quantity of AlF_6_^3−^ present.

While ^27^Al NMR spectroscopy is shown to be a highly sensitive technique for qualitative detection of etched Al, several issues make quantitative detection of the extent of etching difficult. Quadrupolar peak broadening and peak overlap is problematic, both for accurate peak integration and the selection of an appropriate internal standard which does not interfere with the etching byproduct peaks of interest. Alternatively, ^19^F NMR spectroscopy is well suited for quantitative detection as it generally displays sharp peaks and has numerous potential internal standards. Trifluoroacetic acid (TFA), which was shown earlier to be unchanged under etching conditions, was selected as an internal standard. Na_3_AlF_6_ was selected as the analyte for calibration due to the identical nature of its ^19^F NMR peaks with the peaks observed during Ti_3_AlC_2_ MAX phase etching. A calibration curve was prepared by adding TFA (2 mmol) to 10 mL of concentrated aqueous H_2_SiF_6_, followed by measured quantities of Na_3_AlF_6_. ^19^F NMR spectra were recorded following each addition and the ratio of ^19^F NMR peak integrations of AlF_6_^3−^ to TFA were plotted against the added quantity of Na_3_AlF_6_ (Figure 5a). Additional details regarding calibration curve preparation can be found in the supporting information (Table S8).

The prepared calibration curve was then used to monitor a Ti_3_AlC_2_ MAX etching reaction using the optimized H_2_SiF_6_ etching conditions (48 h at 35 °C) with TFA (2 mmol) added as an internal standard. Aliquots of the etching supernatant were examined by ^19^F NMR at times ranging from 4 to 96 h. The ratio of ^19^F NMR peak integrations of AlF_6_^3−^ to TFA contained within the aliquots was then plotted along the linear regression line to reveal the quantity of etched Al present at each time point (Figure 5b). The quantity of AlF_6_^3−^ contained in the etching supernatant, calculated from the AlF_6_^3−^ to TFA ratios, is seen to increase over the successive timepoints. The calculated quantity of AlF_6_^3−^ stops before reaching the theoretical maximum, dictated by the quantity of Ti_3_AlC_2_ MAX added to the etching reaction, leveling off after 68 h at ∼76–80% of the theoretical maximum. The calculated quantity of Al etched over the approximate time period (∼44 h) of the optimized reaction conditions was found to be ∼46%. This value aligns well with the synthesis yield (∼25 ± 3%) obtained from the optimized H_2_SiF_6_ etching protocol when accounting for yield losses during delamination and isolation of the single‐ to few‐layer Ti_3_C_2_T*_x_* MXene. Additional details regarding the etching monitoring experiment using ^19^F NMR can be found in the supporting information (Table S9). These results demonstrate the feasibility of using ^19^F NMR spectroscopy as a technique for quantitative detection of etching using H_2_SiF_6_.

Based on the results of the ^27^Al and ^19^F NMR spectroscopic study of the etching process, which point toward H_3_AlF_6_ as the primary etching byproduct, and the earlier described detection of SiO_2_^/^SiO_x_, we propose that the Ti_3_AlC_2_ MAX phase etching process proceeds according to the following overall balanced equation:

(1) $$2Ti_{3}\mathrm{Al}C_{2}+2H_{2}\mathrm{Si}F_{6}+4H_{2}O\to 2Ti_{3}C_{2}+2H_{3}\mathrm{Al}F_{6}+3H_{2}+2\mathrm{Si}O_{2}$$

Presumably there are several intermediate steps involved in the overall reaction, including a first (strong) and second (weak) acid dissociation from H_2_SiF_6_, Equations (2), (3) respectively. The protons released in these dissociations enable proton‐mediated weakening of the Ti‐Al bond contained in the Ti_3_AlC_2_ MAX phase.

(2)

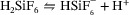

(3) $${\mathrm{HSiF}}_{6}^{-}⇋{\mathrm{SiF}}_{6}^{2-}+H^{+}$$

Following the dissociation of the acid, Ti_3_AlC_2_ MAX phase may be etched by the generated SiF_6_^2−^ or SiF_6_^2−^ may further dissociate (Equation 4) to generate free fluoride (F^−^) as the etchant.

(4) $${\mathrm{SiF}}_{6}^{2-}⇋\mathrm{Si}F_{4}+2\;F^{-}$$

The equilibrium position of Equation 4 and the time it takes to reach that equilibrium are influenced by both the concentration and pH of the solution. The values of those two factors within the H_2_SiF_6_ etching system highly favor the hexafluorosilicate species (SiF_6_^2−^) [43]. This is supported by the absence of free fluoride, as determined using ^19^F NMR spectroscopy, in any of the reactions using H_2_SiF_6_. Any free fluoride which is produced is below the detection limit of ^19^F NMR spectroscopy, indicating that if free fluoride is playing a mechanistic role enabling MAX phase etching, it is released from H_2_SiF_6_ in a highly controlled manner.

Following generation of the active etchant, and activation of the Ti_3_AlC_2_ MAX phase toward etching via proton‐mediated weakening of the Ti─Al bond, the proposed intermediate reaction equation:

(5) $$2Ti_{3}\mathrm{Al}C_{2}+3H_{2}\mathrm{Si}F_{6}\to 2\;\mathrm{Al}F_{3}+2\;Ti_{3}C_{2}+3\;H_{2}+3\;\mathrm{Si}F_{4}$$

leads to the formation of both AlF_3_ and SiF_4_ as intermediate byproducts. These intermediate byproducts are subsequently converted to the byproducts shown in the original Equation 1. AlF_3_ can react with additional H_2_SiF_6_ according to the equation:

(6) $$2\;\mathrm{Al}F_{3}+3\;H_{2}\mathrm{Si}F_{6}\to 2\;H_{3}\mathrm{Al}F_{6}+3\;\mathrm{Si}F_{4}$$

and SiF_4_ can react with water according to the equation:

(7) $$3\;\mathrm{Si}F_{4}+2\;H_{2}O\to 2\;H_{2}\mathrm{Si}F_{6}+\mathrm{Si}O_{2}$$

to produce SiO_2_. When combined, the intermediate reactions Equations (5), (6), (7) yield the original overall balanced Equation 1.

## 3 Conclusion

In conclusion, we have introduced ^27^Al NMR spectroscopy as a highly sensitive tool for the detection of aluminum etched from Ti_3_AlC_2_ MAX phase. This process will allow the rapid exploration of new potential etching systems without strict reliance on sophisticated characterization techniques like XRD, SEM, EDS, or XPS. We have applied this process to the development of a new protocol for selectively etching Ti_3_AlC_2_ MAX using H_2_SiF_6_, producing single‐to‐few‐layered Ti_3_C_2_T*_x_* MXene with ∼25% synthesis yield and ∼7000 S/cm bulk electrical conductivity of the Ti_3_C_2_T*_x_* MXene film. We have demonstrated this process to be free of HF or free fluoride in quantities detectable by ^19^F NMR spectroscopic analysis, although free fluoride may still play a mechanistic role. ^19^F NMR spectroscopy was additionally applied to the quantitative determination of the extent of etching of Ti_3_AlC_2_ MAX phase by H_2_SiF_6_.

## Conflicts of Interest

The authors declare no conflicts of interest.

## Supporting information




**Supporting File**: smtd70455‐sup‐0001‐SuppMat.pdf.

## Data Availability

The data that support the findings of this study are available in the supplementary material of this article.
