# Supplementary material for: NMR Spectroscopy for Studying the Selective Etching of Ti3AlC2 to Ti3C2T x MXene Using Hexafluorosilicic Acid
Source: Small Methods. 2026 Jan 4;10(3):e01640. doi: 10.1002/smtd.202501640 (PMC12893260; doi:10.1002/smtd.202501640)
Supplement: Supplementary file 1 — Supporting File: smtd70455‐sup‐0001‐SuppMat.pdf. [file SMTD-10-e01640-s001.pdf]

SUPPORTING INFORMATION

NMR spectroscopy for studying the selective etching of Ti_3AlC_2 to $Ti_3C_2T_x$ MXene using hexafluorosilicic acid

Henry J. Hamann[a], Anupma Thakur[b], Nithin Chandran B. S. [b], Krutarth Kamath,[b] Babak Anasori[b,c]*, and P. Veeraraghavan Ramachandran[a]*

[a] Department of Chemistry, Purdue University, West Lafayette, Indiana 47907, United States
[b] School of Materials Engineering, Purdue University, West Lafayette, Indiana 47907, United States
[c] School of Mechanical Engineering, Purdue University, West Lafayette, Indiana 47907, United States

*E-mail: chandran@purdue.edu; banasori@purdue.edu

Contents:

Page

General information S3-S5

Initial ^{27}Al NMR of HF/HCl MAX phase etching.. S6

Carboxylic acid etching experimental data S7-S13

Optimization of hexafluorosilicic acid etching of Ti_3AlC_2 MAX phase S14-S23

^{27}Al and ^{19}F NMR spectroscopy study of the H_2SiF_6 etching protocol S24-S26

^{19}F NMR study on the effect of Al concentration in HF solution S27

Comparison of XRD patterns from H_2SiF_6 etching byproducts with standards................ S28-S31

Preparation of calibration curve and etching reaction monitoring by ^{19}F NMR S32-S33

References S34

General Information

Chemical sources

Trifluoroacetic acid, oxalic acid, hexafluorosilicic acid, hexafluorotitanic acid, hexafluorozirconic acid, and hydrofluoric acid were purchased from Sigma-Aldrich (St. Louis, USA), Oakwood (Estill, USA) or Fisher Scientific (Waltham, USA) and used as received. Deionized (DI) water was supplied from in-house tap. Aluminum powder, aluminum trifluoride, and aluminum oxide were purchased from the same commercial sources and used as received. Ti_3AlC_2 MAX phase was prepared from titanium powder (325 mesh, 99.5%), titanium carbide powder (99.5%) and aluminum powder (325 mesh, 99.5%) purchased from Fisher Scientific, USA, using the procedure described below. The hydrofluoric acid (HF, 48-51% solution in water) was purchased from Across Organics. Lithium chloride (LiCl, 98% grade, Thermo Scientific) and hydrochloric acid (HCl, 12 M) were purchased from Fisher Scientific and used as received.

Nuclear magnetic resonance spectroscopy

The nuclear magnetic resonance (NMR) spectra were recorded from a Bruker (Billerica, USA) 400 MHz and chemical shift values (δ) measured in parts per million (ppm). The ^{27}Al NMR (104 MHz) spectra were recorded at ambient temperature and chemical shifts were reported relative to the external standard, $AlCl_3$ in D_2O ($Al(D_2O)_6Cl_3$) (δ = 0 ppm). ^{19}F NMR (376 MHz) spectra were recorded at ambient temperature (299 K) and chemical shifts are reported relative to the external standard $CFCl_3$ (δ = 0 ppm). Quantitative studies used trifluoroacetic acid (TFA) as an internal standard (δ = -76.55 ppm) referenced to external standard $CFCl_3$. ^{19}F NMR (376 MHz) spectra were recorded with a pulse width of 6.0000 μs and a relaxation delay of 2.000 s. ^{19}F NMR spectra for quantitative studies were acquired with 64 scans and all non-quantitative studies were acquired with 16 scans. NMR spectra obtained for samples from experiments using carboxylic acids were taken using standard borosilicate glass NMR tubes. NMR spectra obtained for samples from experiments using fluoro- acids were taken using a standard borosilicate NMR tube equipped with a Teflon NMR tube liner.

Ti_3AlC_2 MAX synthesis

Ti_3AlC_2 MAX phase was synthesized using chemical powders of titanium carbide, titanium, and aluminum by mixing in a 2:1.25:2.2 molar ratio. The powders were then jar-milled and then packed into an alumina crucible. The powders were placed in a high-temperature tube furnace (Carbolite Gero, 1700 °C model) for reactive pressureless sintering at 1400 °C at a 3.5 °C/min ramp rate and held for 4 h under a constant argon flow. Then the Ti_3AlC_2 MAX sintered bock was milled to a fine powder, sieved to particle size < 71 μm and used for $Ti_3C_2T_x$ MXene synthesis.

HF/HCl mixed acid etching protocol[1]

Initially, the synthesized Ti_3AlC_2 MAX powder was acid wash before selective etching using 9 M HCl. For 1 g of acid-washed Ti_3AlC_2 MAX powder, 30 mL of etchant was used for the HF/HCl mixed acid etching protocol. The etchant solution was a mixture of 12 M HCl, deionized water, and 28.4 M HF in the 6:3:1 volume ratio and the reaction was carried out at 35 °C for 24 h. After the reaction, etched multilayered $Ti_3C_2T_x$ MXene was washed to neutral pH by repeated centrifugation (4-5 cycles) at 3234 RCF for 5 min.

MXene delamination procedure

The delamination reaction was carried out using 0.47 M LiCl in 50 mL DI water for 1 g of FSA etched $Ti_3C_2T_x$ MXene powder. This reaction was setup for 18 h at 22 °C. The lithium intercalated $Ti_3C_2T_x$ MXene was washed with DI water by repeated centrifugation at 3234 RCF for 5, 10, 15, and 20 minutes. Then the lithium intercalated $Ti_3C_2T_x$ MXene clay was re-dispersed in deionized water and vortexed for 30 minutes, and then $Ti_3C_2T_x$ MXene colloidal suspension was processed by centrifugation at 2380 RCF for 30 minutes to separate single-to-few MXene layers.

Thin-film preparation

$Ti_3C_2T_x$ MXene film was prepared by vacuum-assisted filtration of single-to-few layered MXene colloidal suspension processed at 2380 RCF using a 0.25 μm filter membrane. Then the $Ti_3C_2T_x$ MXene film was annealed in a vacuum oven at 200 °C for 24 h before the material characterization.

XRD sample preparation and instrument information

XRD pattern of the as-synthesized Ti_3AlC_2 MAX and $Ti_3C_2T_x$ MXene powders was measured using a XRDynamic 500 diffractometer from Anton Paar using Cu Kα (λ = 1.5406 Å). The samples for XRD measurements were mounted on the fixed sample stage and scanned from 5° to 70° with a step size of 0.01° and a time per step of 20 s.

SEM/EDS material characterization

Field-emission scanning electron microscopy (FESEM) was performed using a JEOL JSM-7800f FESEM with a lower electron detector at an acceleration voltage of 15 kV to study the flake size morphology. The single-to-few $Ti_3C_2T_x$ MXene solution concentration was maintained at < 0.1 mg/mL and loaded on an anodic disc, followed by vacuum drying for 2 hours. The samples were gold sputtered to reduce the charging and improve the sharpness of SEM images. Energy dispersive X-ray spectroscopy (EDS) measurements on the $Ti_3C_2T_x$ MXene film were conducted using an EDAX octane super detector by zooming in the SEM imaging to 1,000,000x zoom with a 30s exposure time in a point scan. The EDS data was subsequently analyzed using EDAX TEAM software.

Bulk electrical conductivity measurements

The four-point probe connected to a 2400 Keithley Source meter was used to measure the sheet resistance of the $Ti_3C_2T_x$ MXene film. The bulk electrical conductivity of the prepared $Ti_3C_2T_x$ MXene film was measured by using the thickness from cross-sectional SEM image of the $Ti_3C_2T_x$ MXene film and sheet resistance from the four-point probe, using the following equation: $Electrical\ conductivity = \frac{1}{4.532 \times resistance\ (\Omega) \times thickness\ (cm)}$

Raman analysis of $Ti_3C_2T_x$ MXene film and reaction byproduct and instrument information

Raman spectroscopy was performed on a confocal Renishaw InVia (Gloucestershire, U.K.) instrument using a long working distance 50× (NA = 0.5) objective and a Peltier-cooled CCD camera. Measurements on $Ti_3C_2T_x$ films were conducted using a 785 nm diode laser with a 1200 l/mm diffraction grating. Spectral scans were recorded with a 10 s exposure time and 0.94 mW (0.1% power) laser power to ensure no laser-induced disordered carbon was obtained. For reaction byproduct measurements, a 532 nm laser with a 2400 l/mm diffraction grating was used, with spectral scans at a 10 s exposure time and 10.5 mW (5% power) laser power, and three accumulations per scan. The Raman spectra obtained were processed in WIRE 4.4 software for cosmic ray removal, baseline correction, and normalization by division with the maximum intensity.

Initial ^{27}Al NMR of HF/HCl MAX phase etching

$$Ti_3AlC_2 \xrightarrow[35\ ^{o}C,\ 24\ h]{HF/HCl} Ti_3C_2T_x + AlF_3 + H_2$$

Scheme S1. General reaction scheme for HF/HCl mixed acid etching of Ti_3AlC_2 MAX phase.

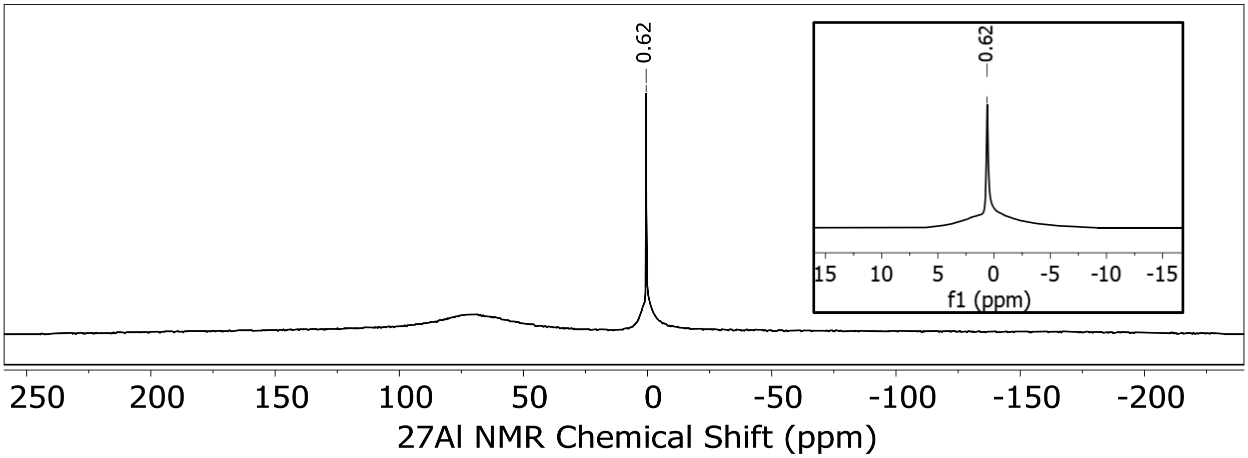

Figure S1. ^{27}Al NMR of supernatant from HF/HCl mixed acid etching of Ti_3AlC_2 MAX phase

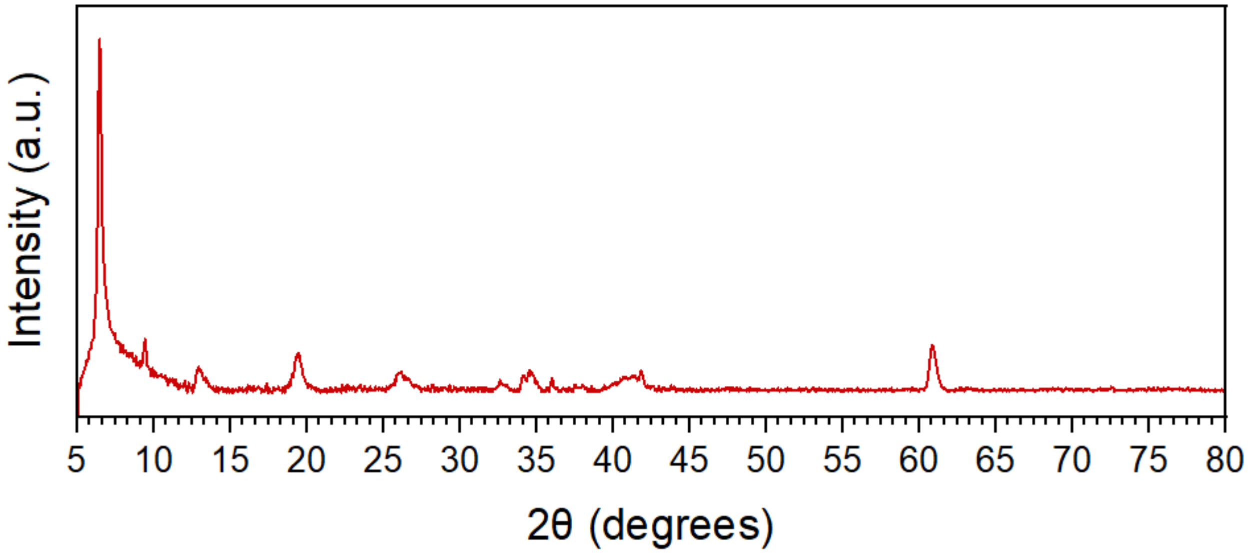

Figure S2. $Ti_3C_2T_x$ MXene XRD pattern obtained from the solid material isolated from HF/HCl mixed acid etching of Ti_3AlC_2 MAX phase.

Carboxylic acid etching experimental data

Procedure for trifluoroacetic acid etching experiments

In a 50 mL plastic centrifuge tube containing a Teflon coated magnetic stir bar was added 7.5 mL of a 50% v/v solution of trifluoroacetic acid in water (~6.5 M). Ti_3AlC_2 MAX phase was weighed (masses listed in Table S1 below) and added with stirring to the aqueous trifluoroacetic acid solution. The centrifuge tube was lightly capped and the stirred suspension was brought to 80 °C using an oil bath. The reactions were stirred for 24 h. After completion of the etching reaction, it was left to come to room temperature, followed by centrifugation. The initial etching supernatant was decanted for analysis by ^{27}Al or ^{19}F NMR spectroscopic analysis. The solid material remaining in the centrifuge tube was resuspended in DI water, centrifuged, and the supernatant decanted 3 times to remove any remaining acid from the etching reaction.
For the reaction performed in the stainless steel autoclave reactor, the 50% v/v trifluoroacetic acid solution in water was added to the autoclaves Teflon liner, followed by the weighed Ti_3AlC_2 MAX phase. The Teflon liner was capped and placed inside of the stainless steel autoclave reactor. The reactor was tightly sealed and placed inside a preheated 90 °C oven, where it was left for 72 h. After completion of the etching reaction, it was removed from the oven and left to come to room temperature, when it was carefully unsealed. The washing and isolation steps remained the same.

Table S1. Conditions tested in trifluoroacetic acid etching experiments.

Entry	Acid[a]	Acid Vol. (mL)[b]	MAX (g)	Temp. (°C)	Time (hours)
1	TFA	7.5	0.25	80	24
2[c]	TFA	7.5	0.25	90	72

[a]TFA = trifluoroacetic acid. [b]A ~6.5 M (50% v/v) aqueous solution of TFA in water was used. [c]Reaction was performed in stainless steel autoclave reactor.

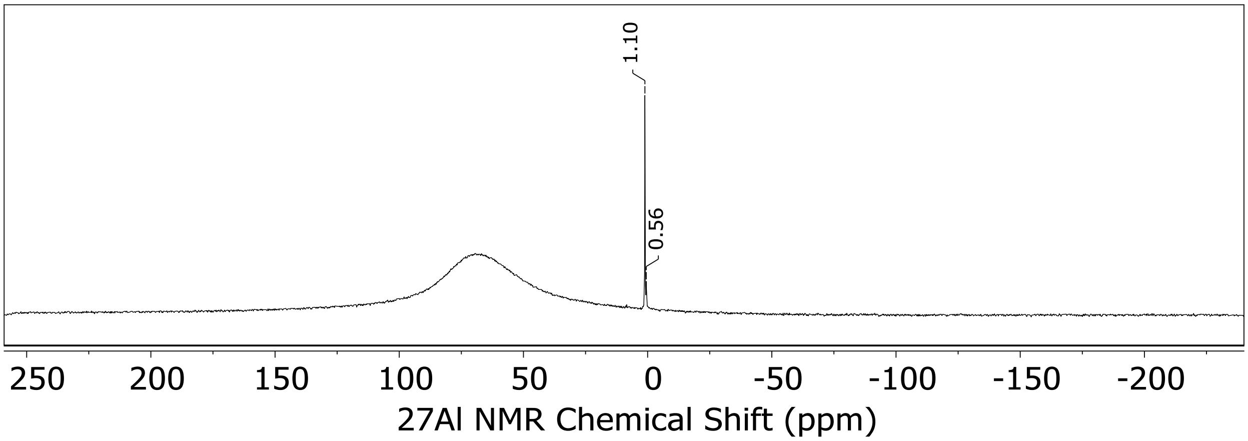

FigureS3. ^{27}Al NMR of supernatant from TFA etching of Ti_3AlC_2 MAX phase (Table S1, Entry 1).

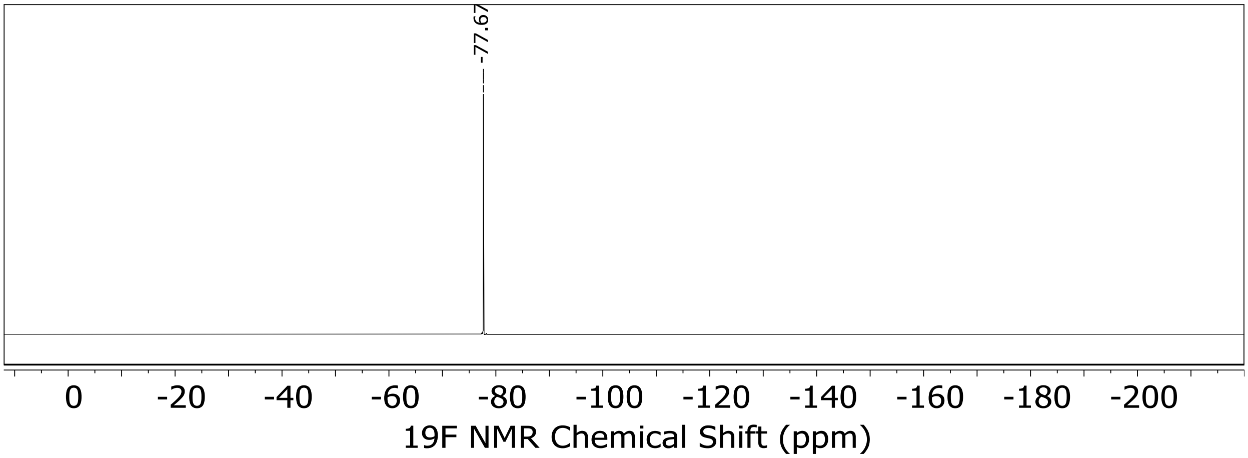

Figure S4. ^{19}F NMR of supernatant from TFA etching of Ti_3AlC_2 MAX phase (Table S1, Entry 1).

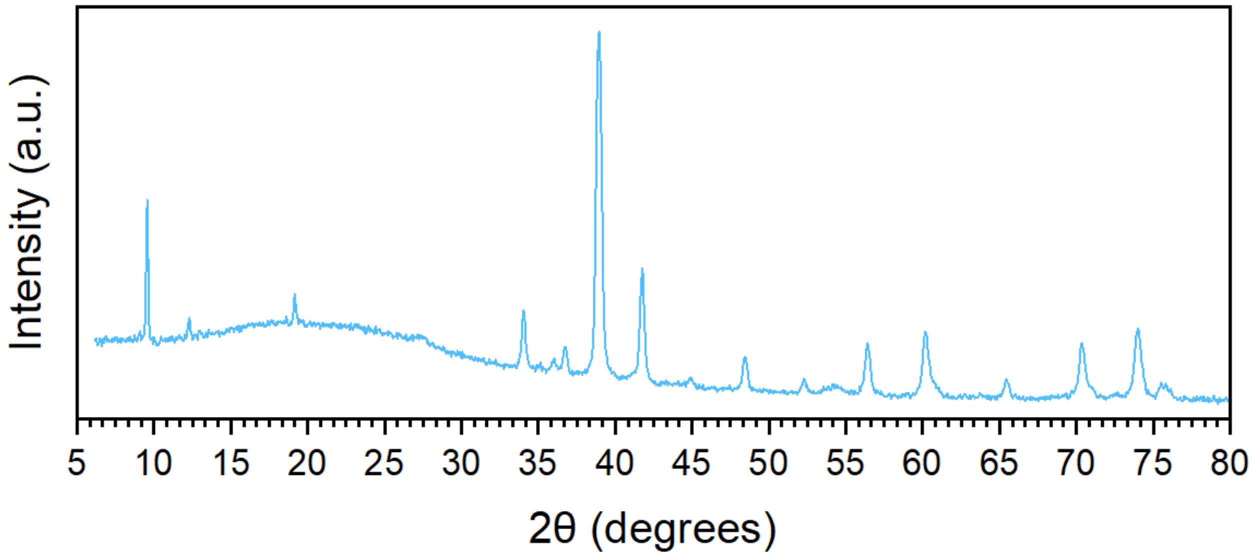

Figure S5. XRD pattern of solid material obtained from TFA etching of Ti_3AlC_2 MAX phase (Table S1, Entry 1).

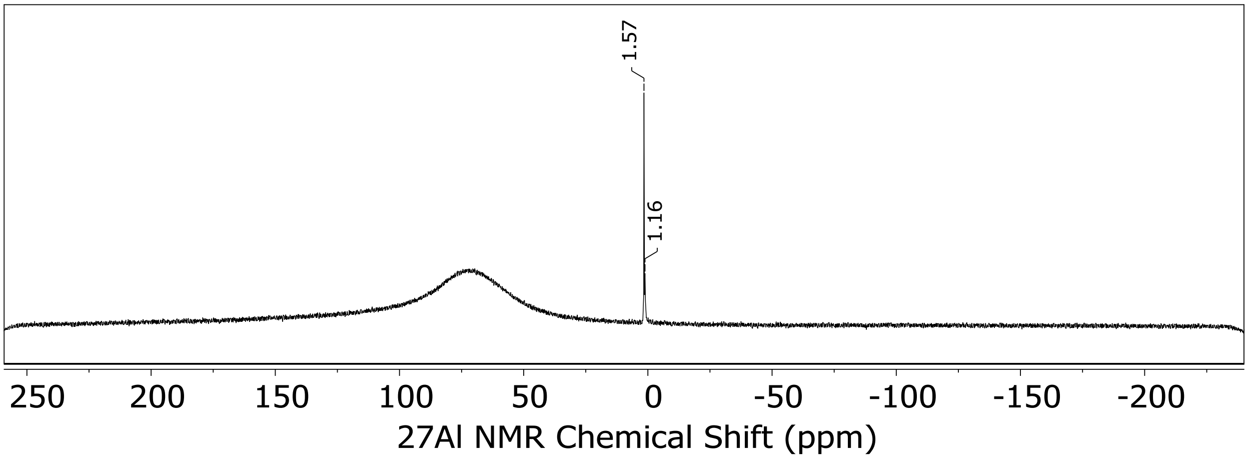

FigureS6. ^{27}Al NMR of supernatant from TFA etching of Ti_3AlC_2 MAX phase (Table S1, Entry 2).

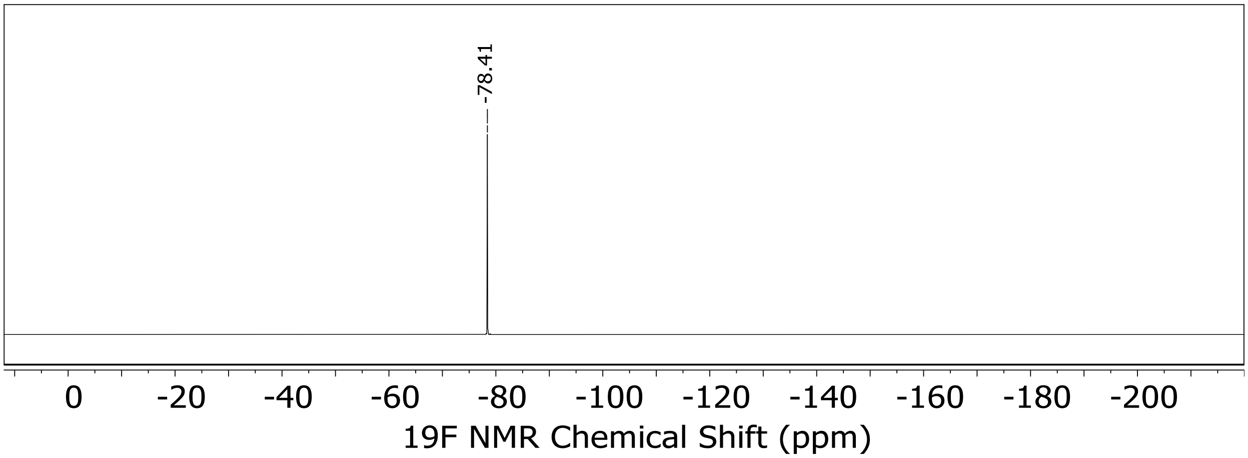

Figure S7. ^{19}F NMR of supernatant from TFA etching of Ti_3AlC_2 MAX phase (Table S1, Entry 2).

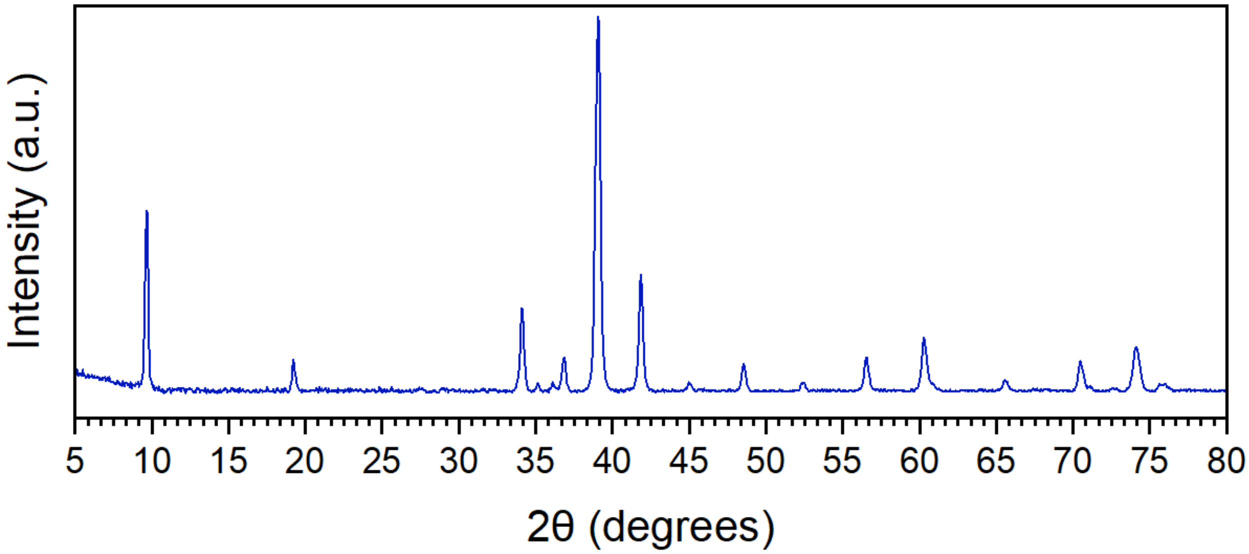

Figure S8. XRD pattern of solid material obtained from TFA etching of Ti_3AlC_2 MAX phase (Table S1, Entry 2).

<u>Procedure for oxalic acid etching experiments</u>

In a 50 mL plastic centrifuge tube containing a Teflon coated magnetic stir bar was added water or acetonitrile (MeCN) (solvent and volumes listed in Table S2 below). Oxalic acid was weighed (masses listed in Table S2 below) and added and dissolved with stirring. Ti_3AlC_2 MAX phase was weighed (masses listed in Table S2 below) and added with stirring to the oxalic acid solution. The centrifuge tube was lightly capped and the stirred suspension was left at room temperature or brought to 50 °C or 100 °C using an oil bath (reaction temperatures are listed in Table S2 below). The reactions were stirred for 24 h. After completion of the etching reaction, it was left to come to room temperature, followed by centrifugation. The initial etching supernatant was decanted for analysis by ^{27}Al NMR spectroscopic analysis. The solid material remaining in the centrifuge tube was resuspended in DI water, centrifuged, and the supernatant decanted 3 times to remove any remaining acid from the etching reaction.

Table S2. Conditions tested in oxalic acid etching experiments.

Entry	Acid	Acid (g)	Solvent	Solvent (mL)	MAX (g)	Temp. (°C)	Time (hours)
1	oxalic	6.75	H_2O	75	1	50	24
2	oxalic	6.75	H_2O	75	1	RT	24
3	oxalic	1.35	H_2O	15	0.5	100	24
4	oxalic	1.35	MeCN	15	0.5	100	24

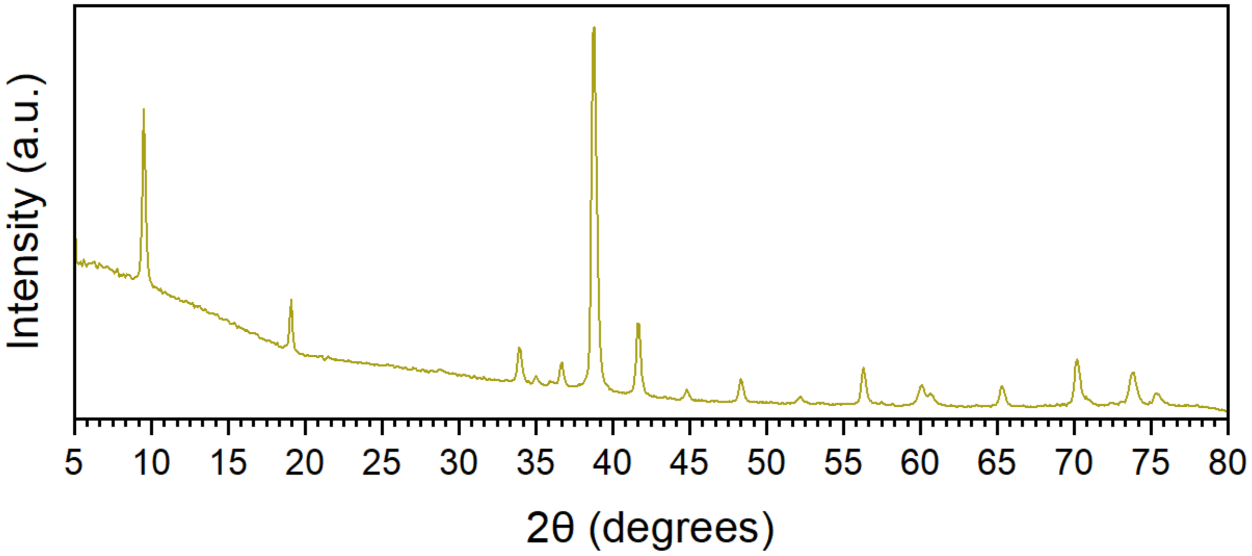

Figure S9. XRD pattern of solid material obtained from oxalic etching of Ti_3AlC_2 MAX phase (Table S2, Entry 1).

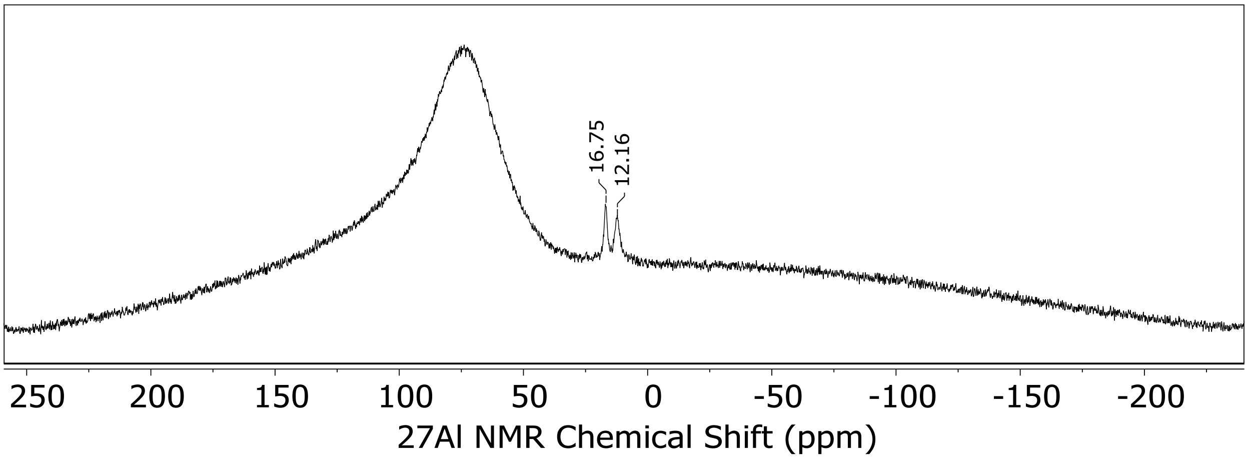

Figure S10. ^{27}Al NMR of supernatant from oxalic etching of Ti_3AlC_2 MAX phase (Table S2, Entry 2).

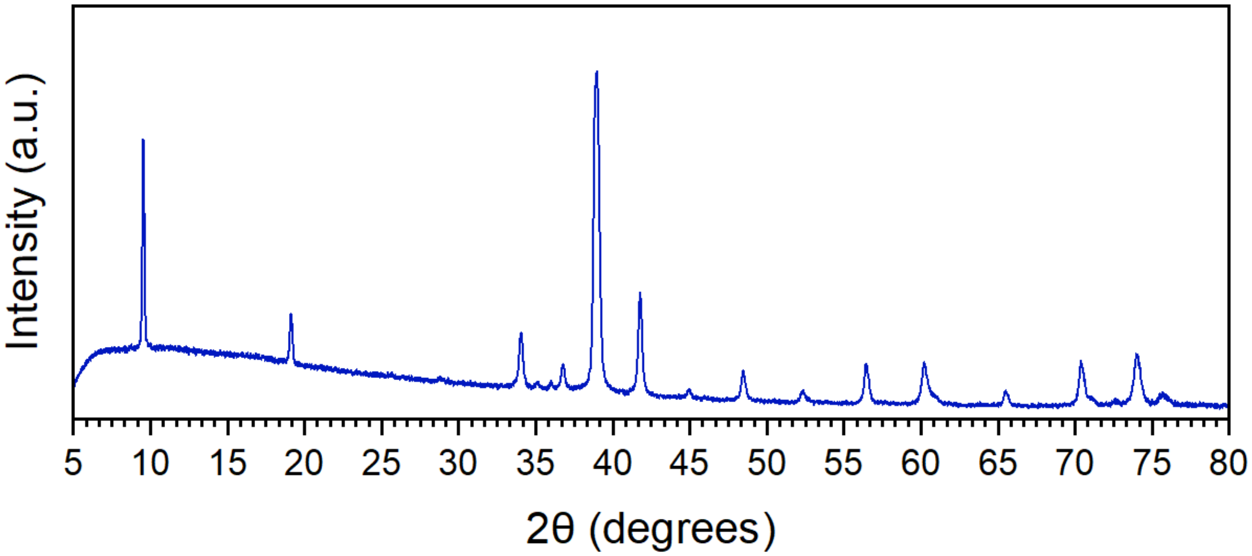

Figure S11. XRD pattern of solid material obtained from oxalic etching of Ti_3AlC_2 MAX phase (Table S2, Entry 2).

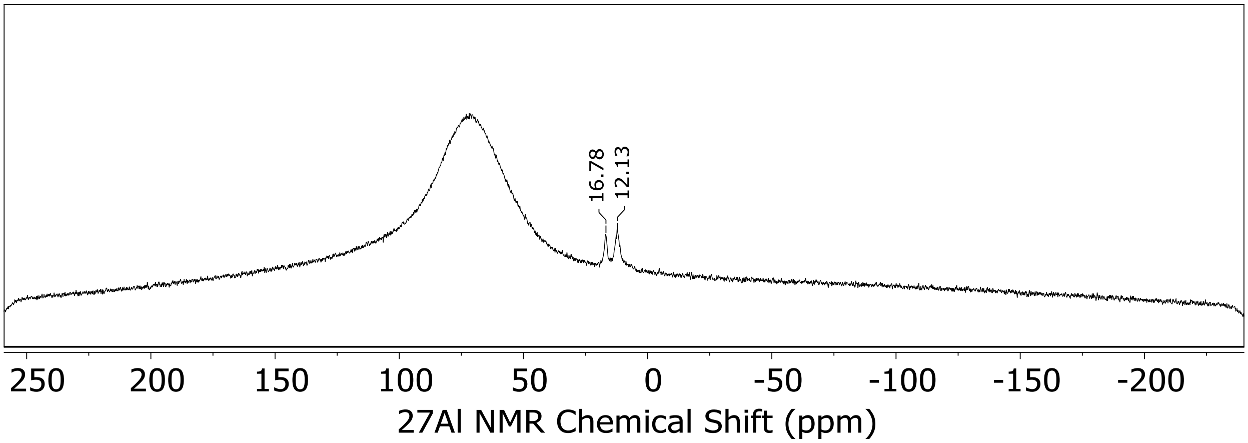

Figure S12. ^{27}Al NMR of supernatant from oxalic etching of Ti_3AlC_2 MAX phase (Table S2, Entry 3).

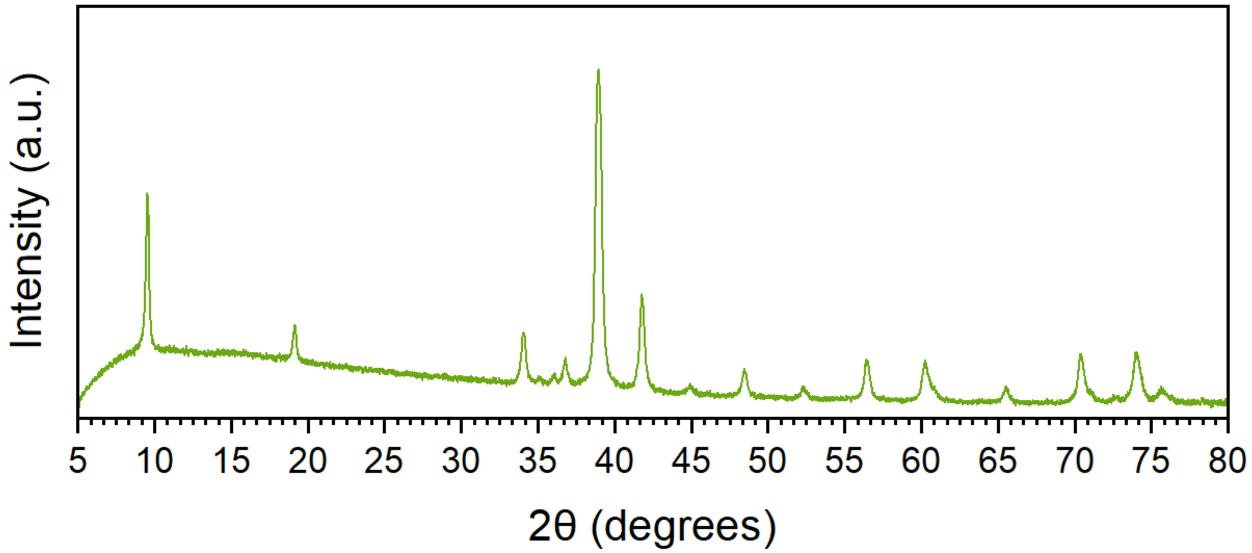

Figure S13. XRD pattern of solid material obtained from oxalic etching of Ti_3AlC_2 MAX phase (Table S2, Entry 3).

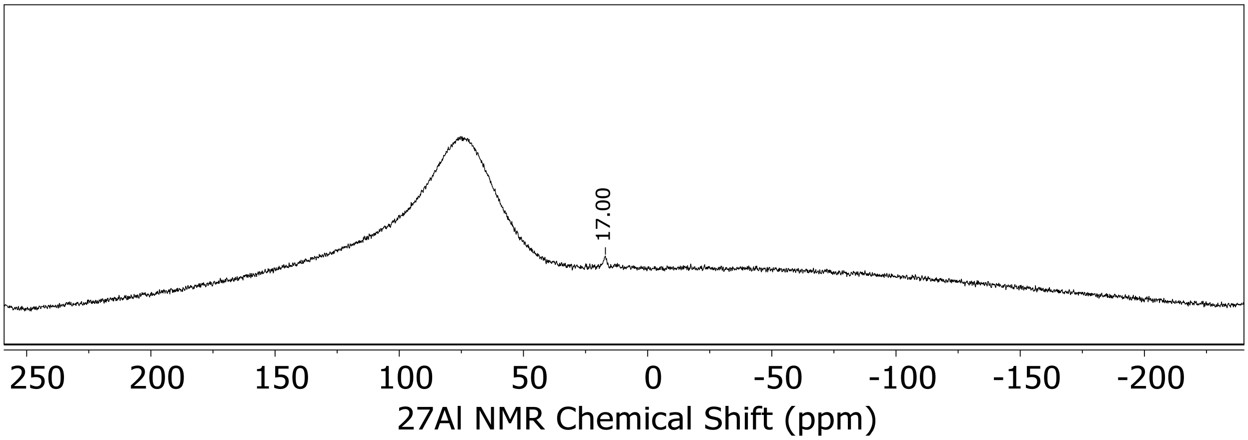

Figure S14. ^{27}Al NMR of supernatant from oxalic etching of Ti_3AlC_2 MAX phase (Table S2, Entry 4).

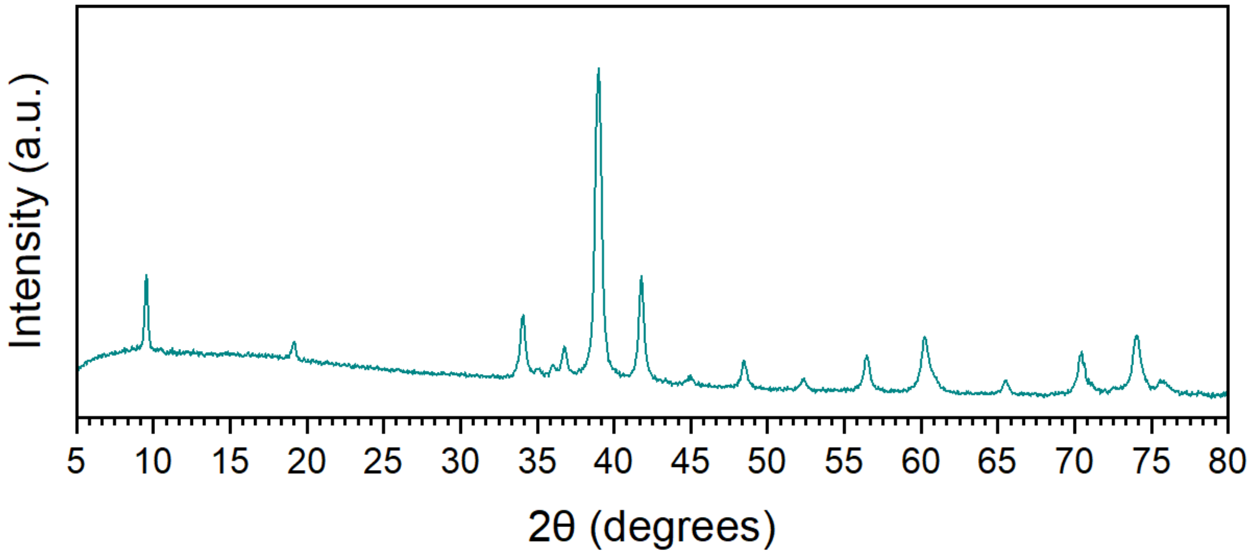

Figure S15. XRD pattern of solid material obtained from oxalic etching of Ti_3AlC_2 MAX phase (Table S2, Entry 4).

Optimization of hexafluorosilicic acid etching of Ti_3AlC_2 MAX phase

$$Ti_3AlC_2 \xrightarrow[35\ ^{o}C,\ 48\ h]{H_2SiF_6} Ti_3C_2T_x + H_3AlF_6 + H_2$$

Scheme S2. General reaction scheme for H_2SiF_6 etching of Ti_3AlC_2 MAX phase.

Procedure for hexafluorosilicic acid etching optimization reactions

In a 50 mL plastic centrifuge tube, a Teflon-coated magnetic stir bar and 35 wt% aqueous hexafluorosilicic acid (volumes listed in Table S3 below) was added. Ti_3AlC_2 MAX phase was weighed (masses listed in Table S3 below) and added with stirring to the aqueous hexafluorosilicic acid solution. The centrifuge tube was lightly capped, and the stirred suspension was left at room temperature or brought to 35 °C or 50 °C using an oil bath (reaction temperatures are listed in Table S3 below). The reactions were stirred for 16 h to 48 h (times listed in Table S3). After completion of the etching reaction, it was left to come to room temperature, followed by centrifugation. The initial etching supernatant was decanted for analysis by ^{27}Al or ^{19}F NMR spectroscopic analysis. The solid material remaining in the centrifuge tube was resuspended in DI water, centrifuged, and the supernatant decanted 3 times to remove any remaining acid from the etching reaction.

Table S3. Table of optimization conditions for hexafluorosilicic acid etching of Ti_3AlC_2 MAX phase.

Entry	Acid	Acid Vol. (mL)	MAX (g)	Temp. (°C)	Time (hours)
1	H_2O	10	0.25	50	48
2	H_2SiF_6	10	0.25	RT	16
3	H_2SiF_6	20	0.25	RT	48
4	H_2SiF_6	10	0.5	RT	48
5	H_2SiF_6	10	0.25	35	24
6	H_2SiF_6	10	0.5	35	24
7	H_2SiF_6	10	0.25	RT	48
8	H_2SiF_6	10	0.25	50	24
9	H_2SiF_6	10	0.5	50	48
10	H_2SiF_6	10	0.25	50	48
**11**	**H_2SiF_6**	**10**	**0.25**	**35**	**48**

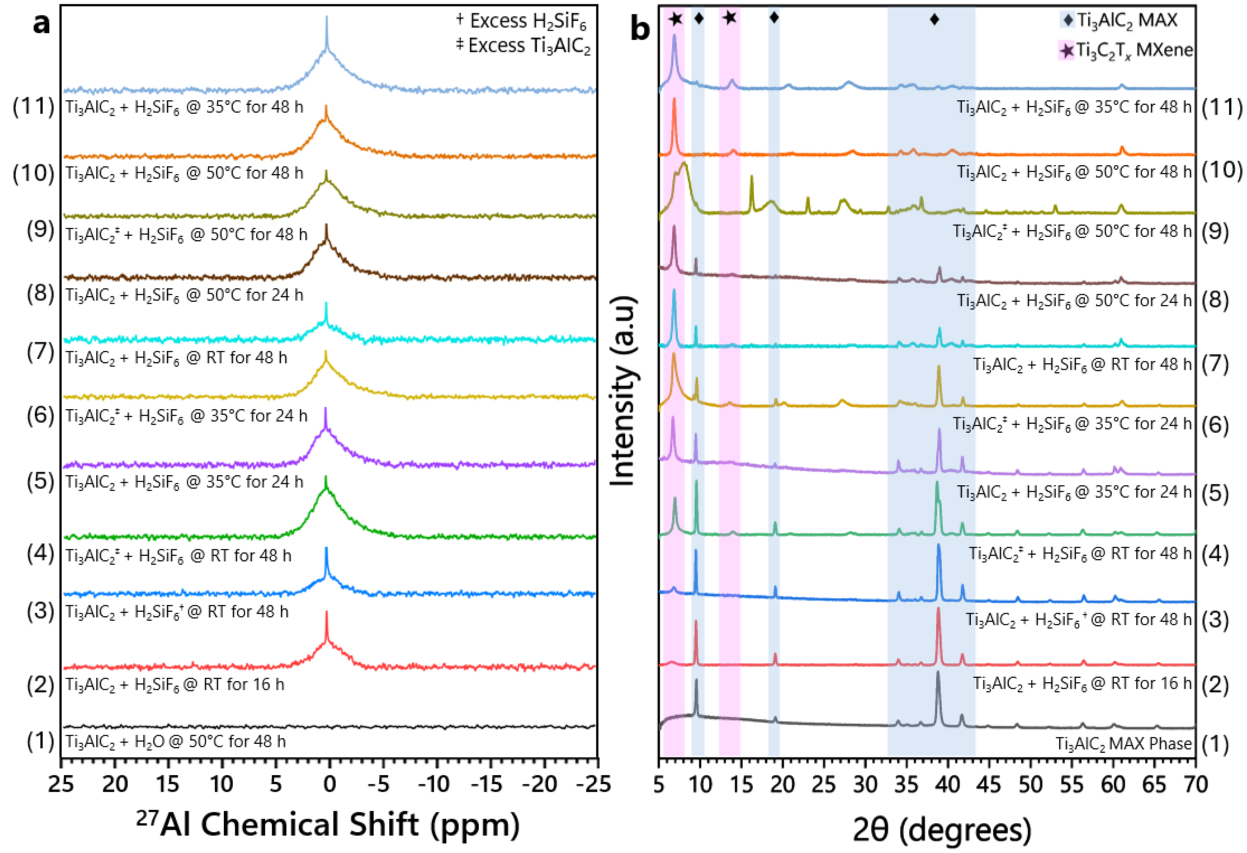

Figure S16. Spectral analysis from optimization of Ti_3AlC_2 etching using H_2SiF_6. Etching reactions used 10 mL of H_2SiF_6 and 0.25 g of Ti_3AlC_2. (a) ^{27}Al NMR spectra obtained from optimization experiments (b) X-ray diffraction patterns obtained from the isolated reaction material. †Used 20 mL H_2SiF_6. ‡Used 0.5 g Ti_3AlC_2.

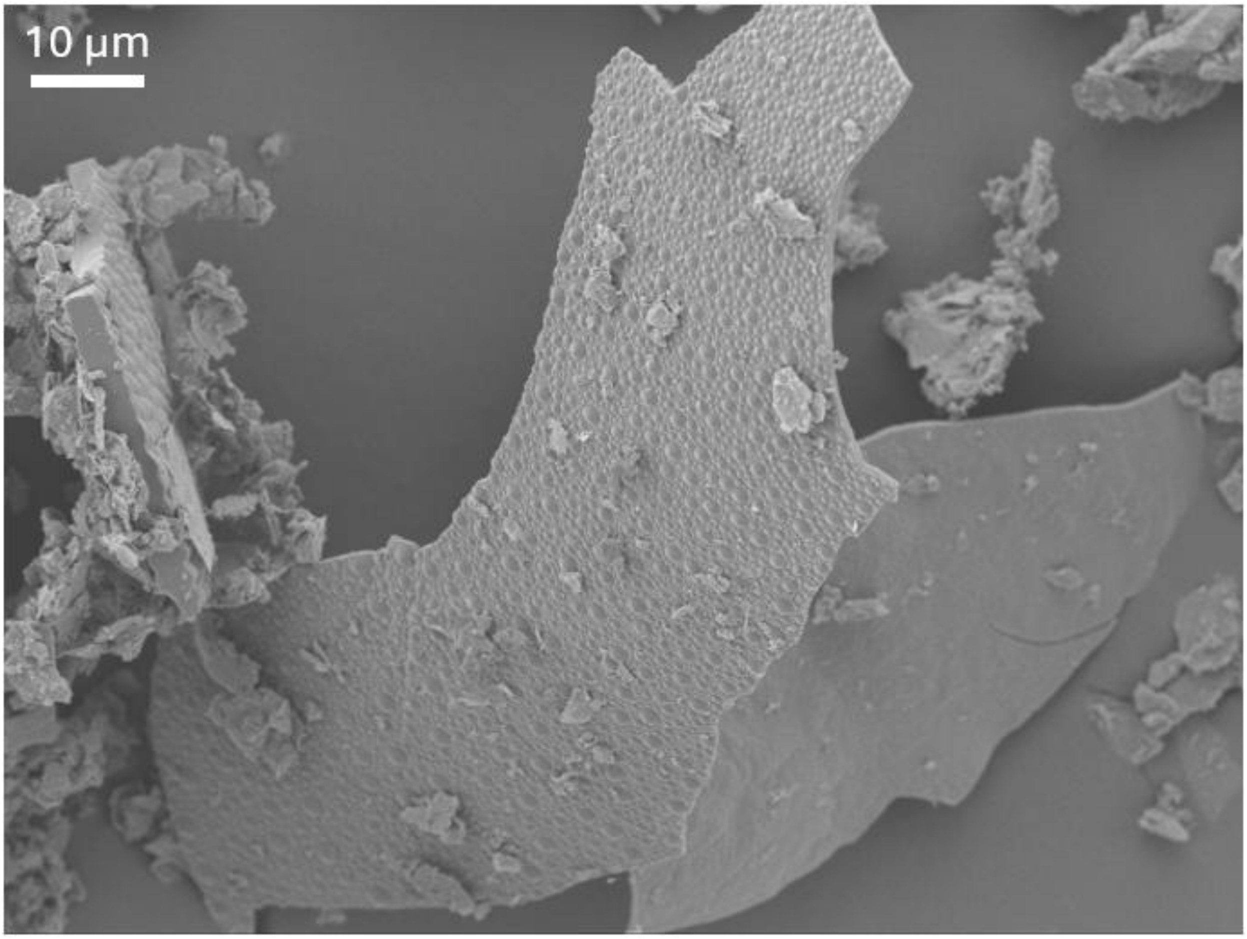

Figure S17. SEM image of flake observed in reactions using hexafluorosilicic acid for etching of Ti_3AlC_2 MAX phase conducted at 50 °C (Table S3, entries 8-10).

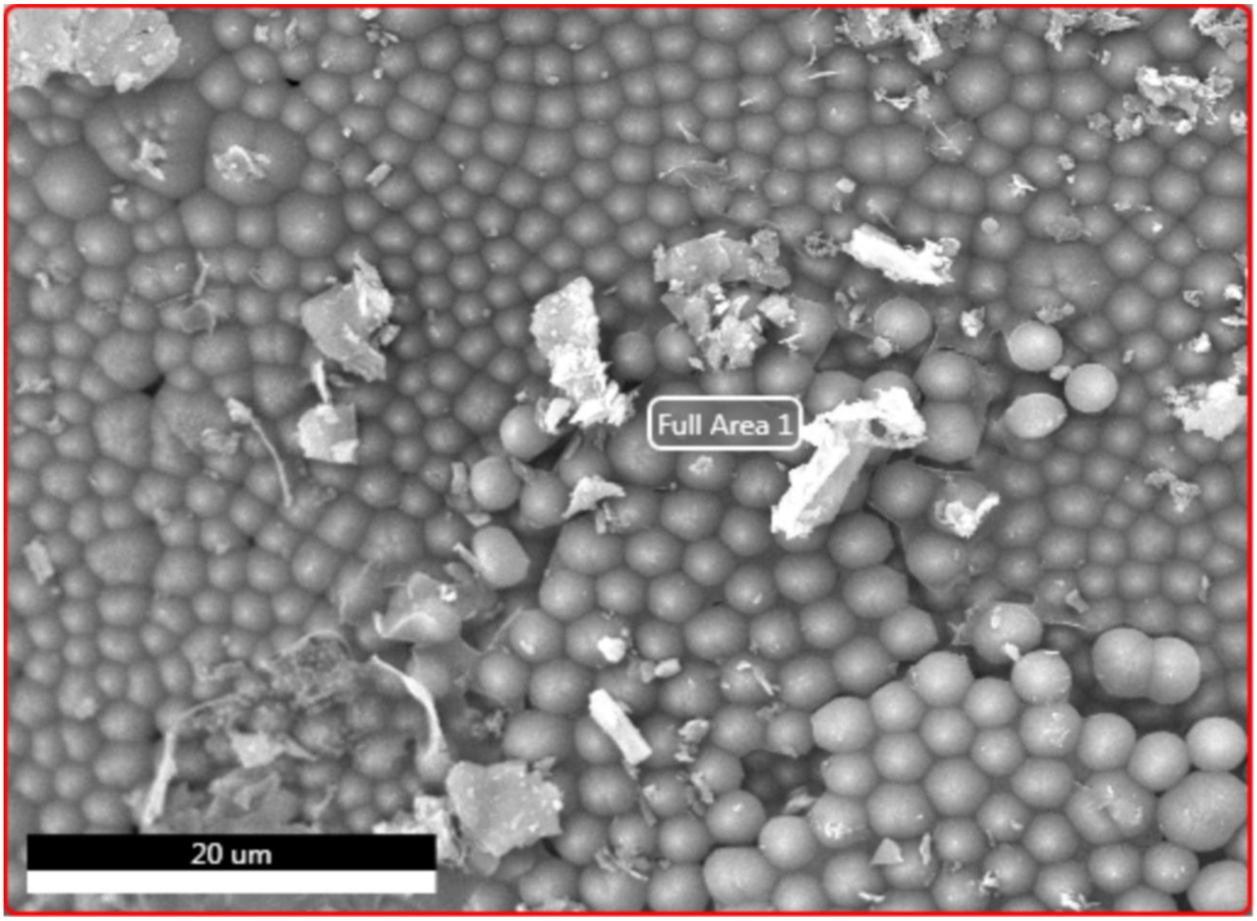

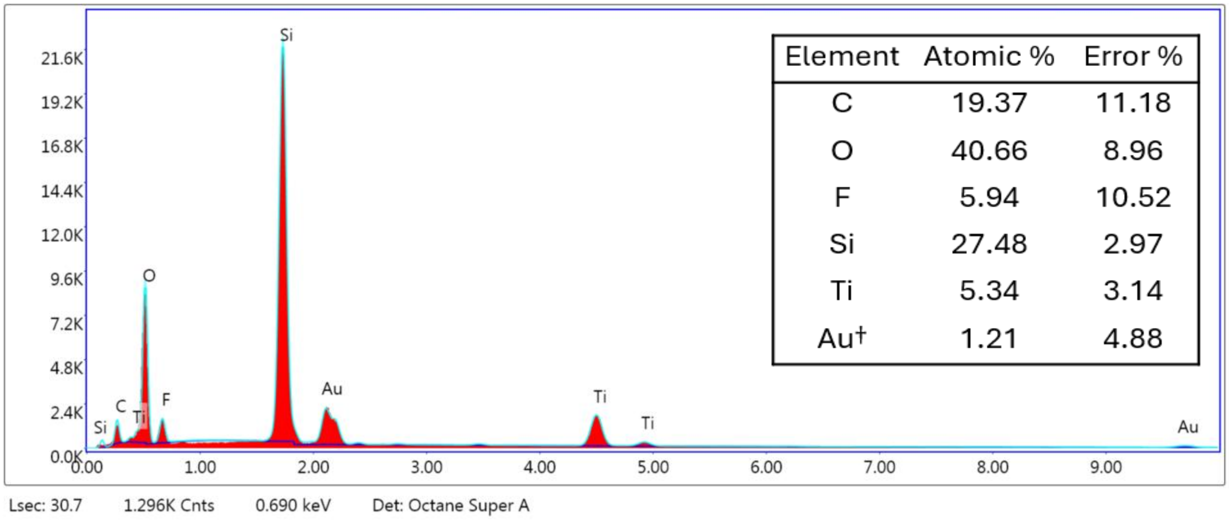

Element	Atomic %	Error %
C	19.37	11.18
O	40.66	8.96
F	5.94	10.52
Si	27.48	2.97
Ti	5.34	3.14
Au†	1.21	4.88

Figure S18. EDS analysis of flake observed in reactions using hexafluorosilicic acid for etching of Ti_3AlC_2 MAX phase conducted at 50 °C (Table S3, entries 8-10). (a) SEM image of the area of EDS analysis. (b) EDS spectrum obtained from the flake and the measured elemental composition. †Gold (Au) is present as result of sputtering during sample preparation.

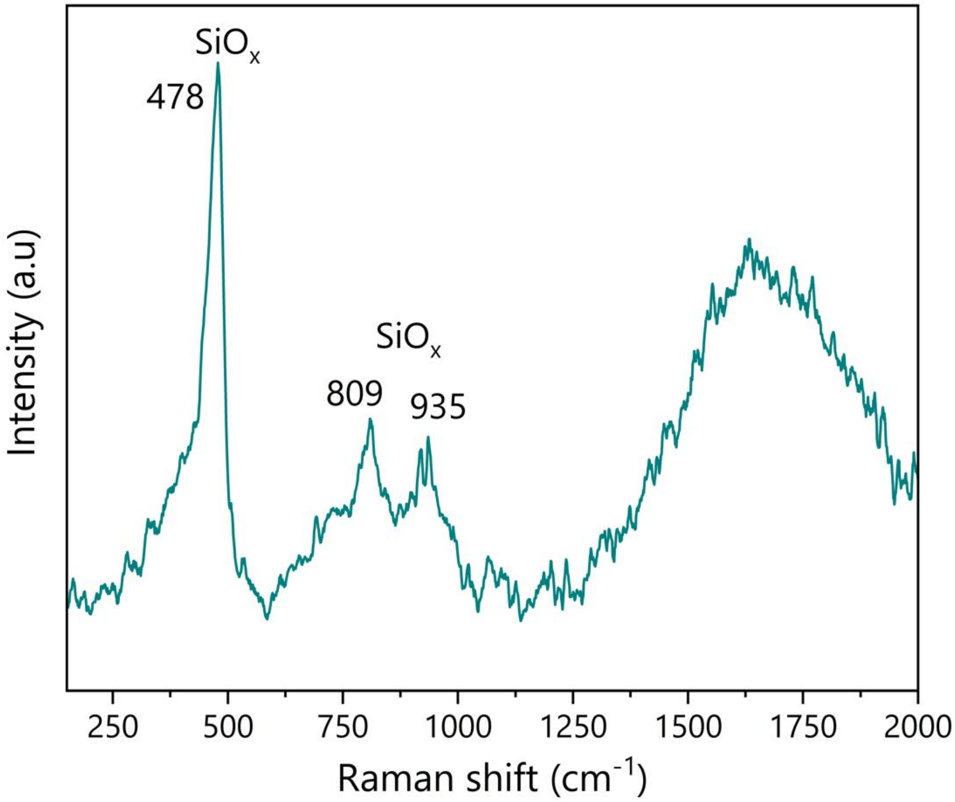

Figure S19. Raman spectra of the flake byproduct acquired using 532 nm laser.

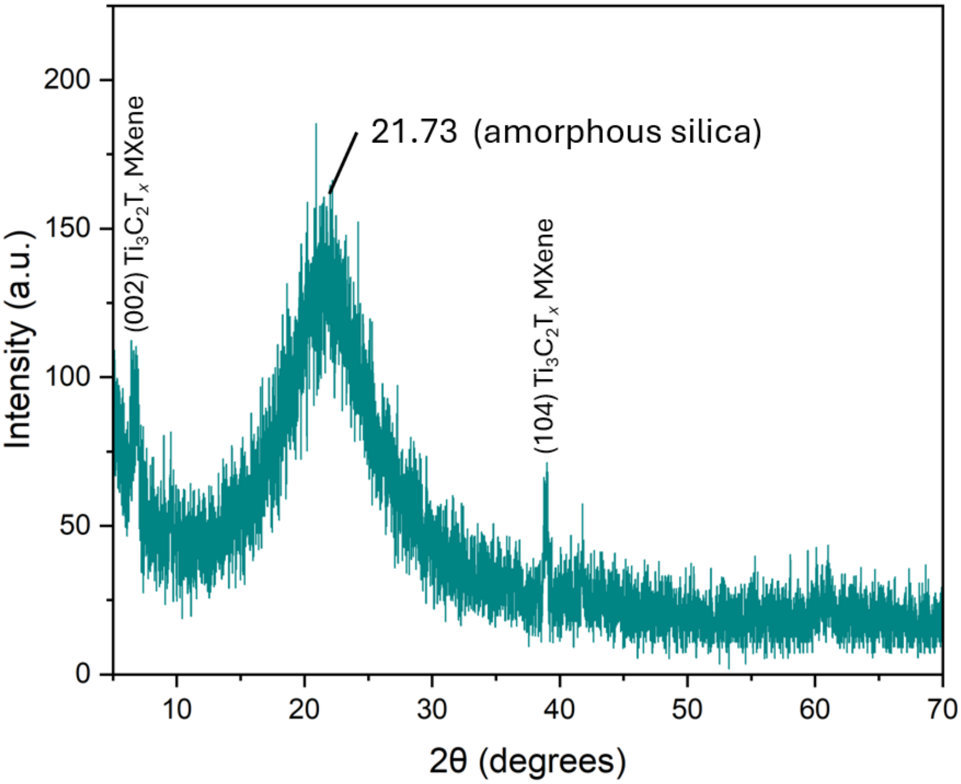

Figure S20. XRD spectra of the flake byproduct confirms the presence of amorphous silica.

Procedure for hexafluorotitanic and hexafluorozirconic acid etching optimization reactions

The above procedure for the hexafluorosilicic acid etching optimization reactions was used with the replacement of hexafluorosilicic acid with either hexafluorotitanic acid or hexafluorozirconic acid. The washing and isolation steps remained the same.

Table S4. Conditions examined for hexafluorotitanic and hexafluorozirconic acid etching optimization reactions

Entry	Acid	Acid Vol. (mL)	MAX (g)	Temp. (°C)	Time (hours)
1	H_2TiF_6	5	0.25	RT	24
2	H_2TiF_6	5	0.25	RT	48
3	H_2ZrF_6	5	0.25	RT	24
4	H_2ZrF_6	5	0.25	RT	48

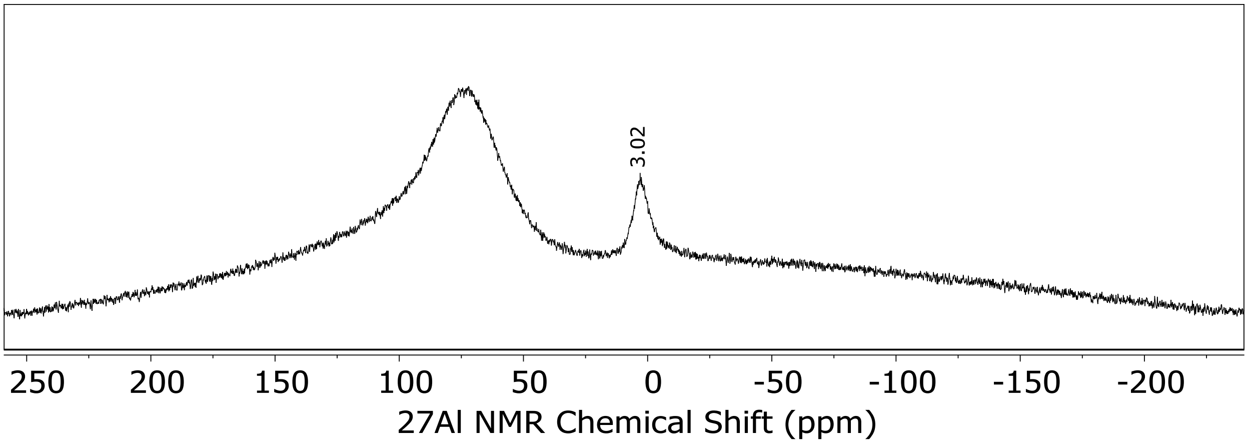

Figure S21. ^{27}Al NMR of supernatant from hexafluorotitanic acid etching of Ti_3AlC_2 MAX phase (Table S4, Entry 1).

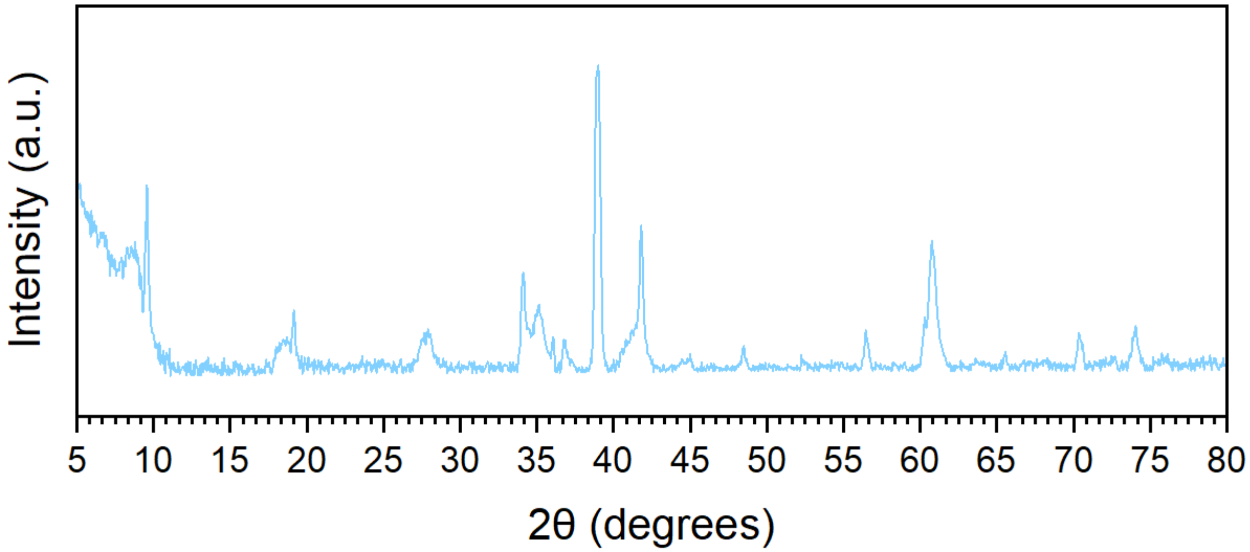

Figure S22. XRD pattern of solid material obtained from hexafluorotitanic acid etching of Ti_3AlC_2 MAX phase (Table S4, Entry 2).

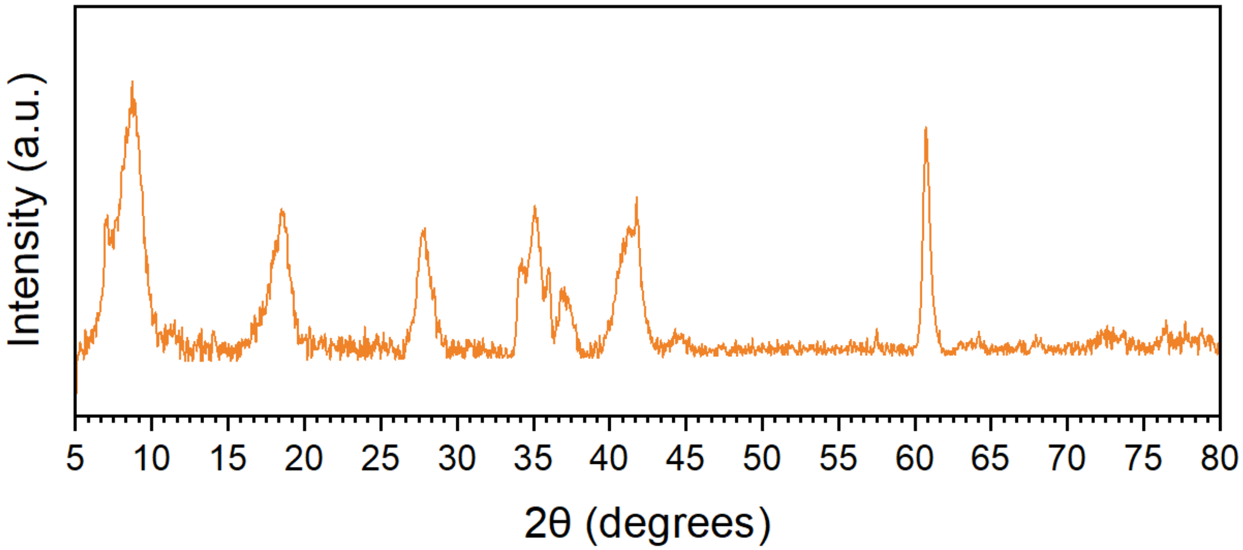

Figure S23. XRD pattern of solid material obtained from hexafluorotitanic acid etching of Ti_3AlC_2 MAX phase (Table S4, Entry 1).

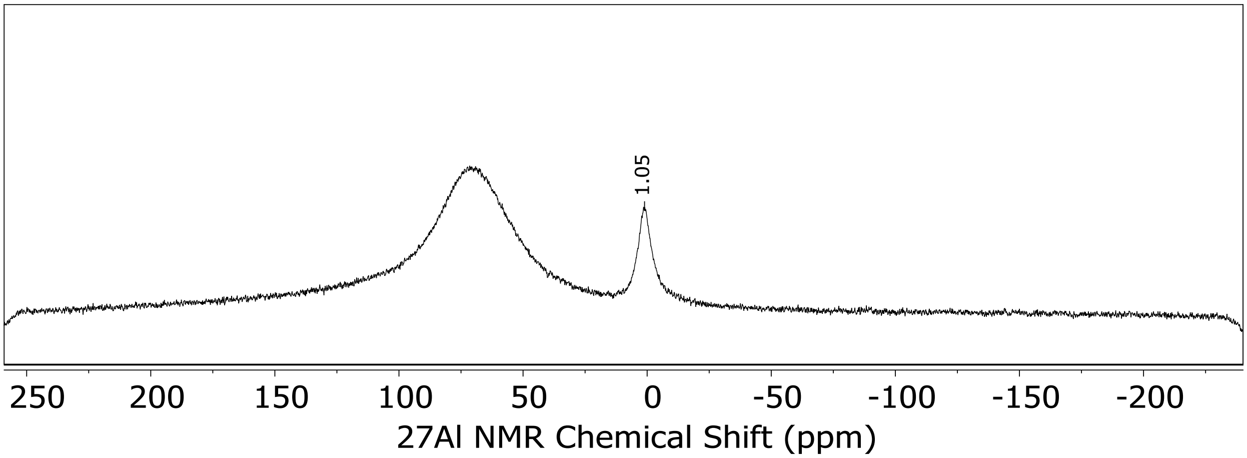

Figure S24. ^{27}Al NMR of supernatant from hexafluorozirconic acid etching of Ti_3AlC_2 MAX phase (Table S4, Entry 3).

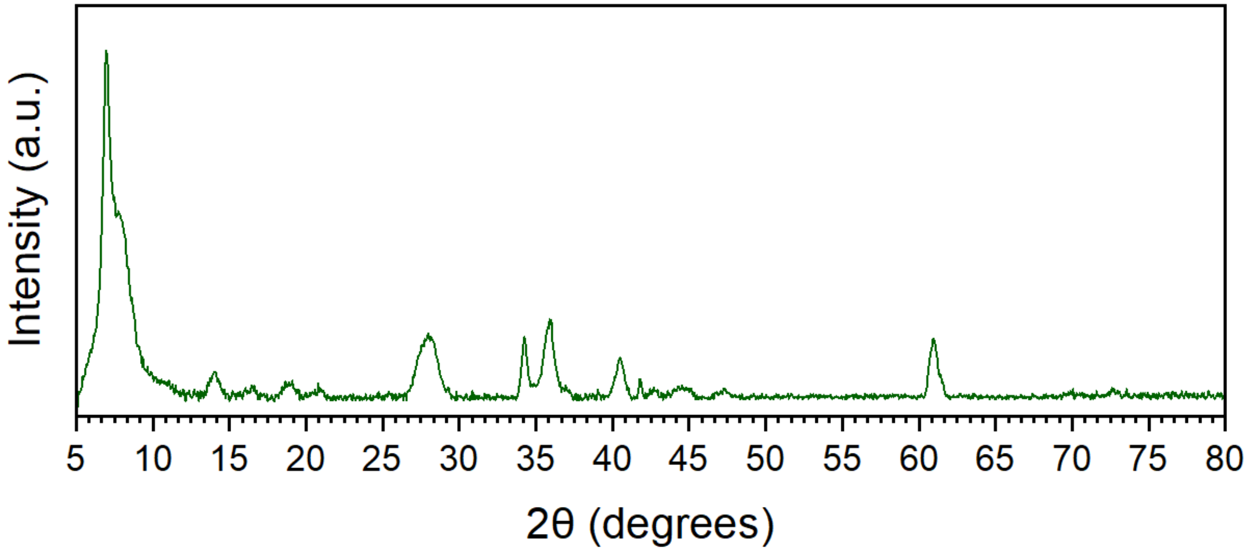

Figure S25. XRD pattern of solid material obtained from hexafluorozirconic acid etching of Ti_3AlC_2 MAX phase (Table S4, Entry 4).

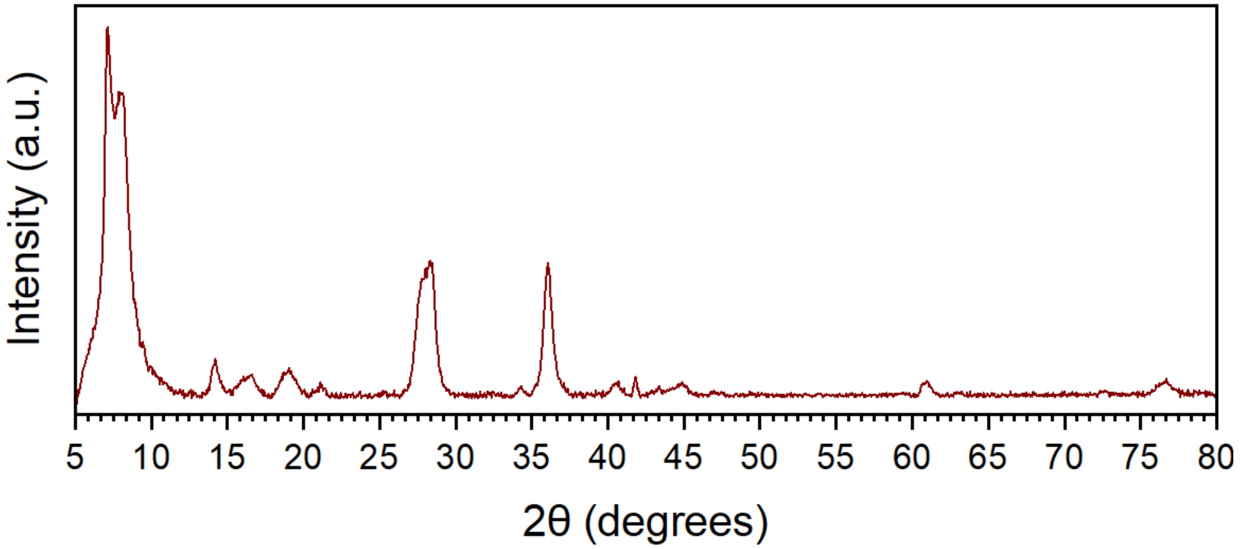

Figure S26. XRD pattern of solid material obtained from hexafluorozirconic acid etching of Ti_3AlC_2 MAX phase (Table S4, Entry 3).

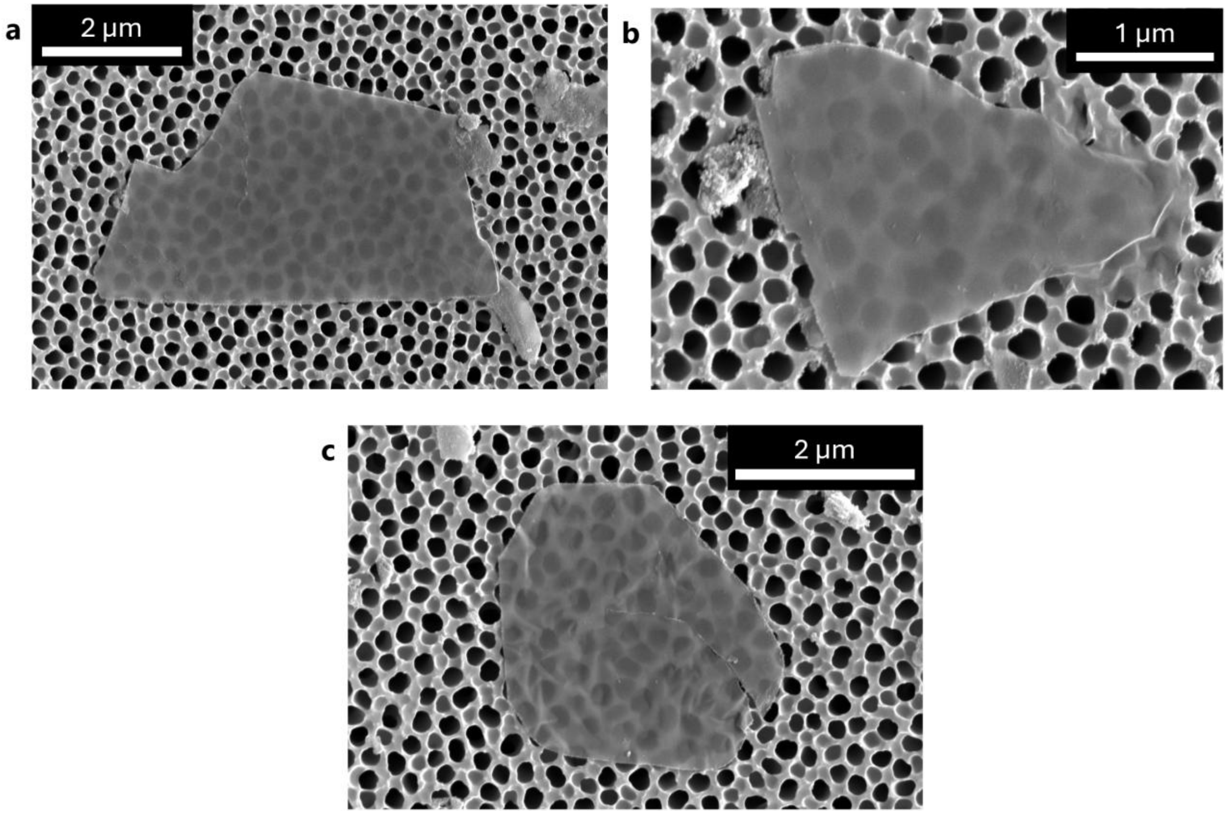

Figure S27. SEM images of the single-to-few-layered $Ti_3C_2T_x$ MXene suspension showing electron-transparent flakes with no visible holes and defects within the 2D flakes.

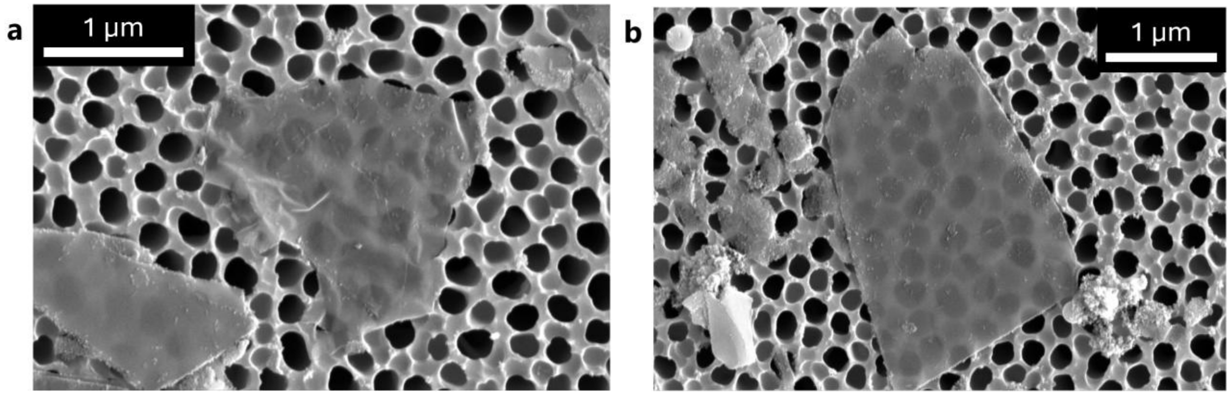

Figure S28. SEM images of the single-to-few-layered $Ti_3C_2T_x$ MXene suspension showing the presence of nanoparticles on the flake surface, residual from the synthesis. As oxides preferentially grow on defective edges first, these surface impurities can be removed with additional washing steps.

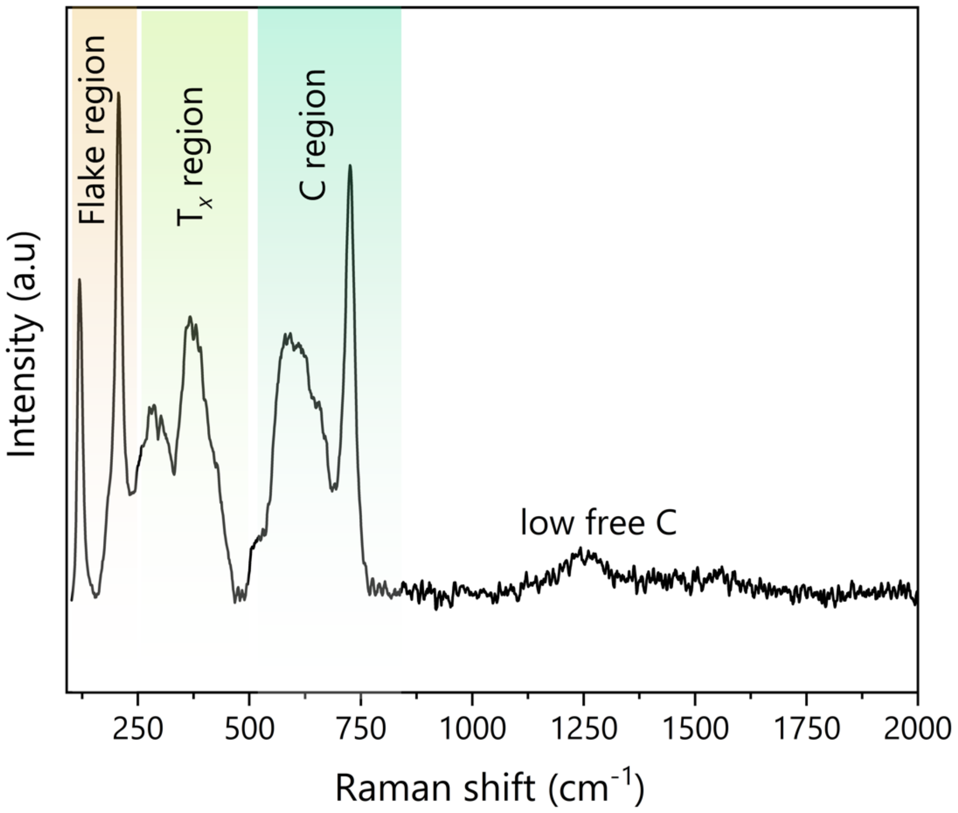

Figure S29. Raman spectra of delaminated $Ti_3C_2T_x$ film acquired using 785 nm laser and 1% power

^{27}Al and ^{19}F NMR spectroscopy study of the H_2SiF_6 etching protocol

Procedure for preparation of AlF_3 and Ti_3AlC_2 standards

In two 15 mL plastic centrifuge tubes each containing a Teflon coated magnetic stir bar was added 5 mL of DI water. To the first tube was added ~100 mg aluminum trifluoride (AlF_3). To the second tube was added ~100 mg Ti_3AlC_2 MAX phase. Each of the tubes was lightly capped and left to stir overnight. An aliquot of each solution was transferred via plastic pipette to a separate Teflon NMR tube liner for analysis by ^{19}F and ^{27}Al NMR spectroscopy.

An aliquot of the aqueous HF was transferred via plastic pipette to a Teflon NMR tube liner for initial analysis. After analysis by ^{19}F NMR spectroscopy, the aliquot was returned to the plastic centrifuge tube. Aluminum foil was weighed (14 mg) and dissolved with stirring in the aqueous HF. After analysis by ^{19}F NMR spectroscopy, the aliquot was returned to the plastic centrifuge tube. This process was repeated following further additions of 13 mg and 27 mg of aluminum foil.

Procedure for preparation of H_2SiF_6 and HF standards

In a 15 mL plastic centrifuge tube containing a Teflon coated magnetic stir bar was added 5 mL of 35 wt% aqueous hexafluorosilicic acid (H_2SiF_6). An aliquot of the solution was transferred via plastic pipette to a Teflon NMR tube liner for analysis by ^{19}F NMR spectroscopy. After analysis, the aliquot was returned to the plastic centrifuge tube. The H_2SiF_6 solution was then brough to 35 °C using an oil bath and stirred for ~12 h. The analysis procedure was repeated and the aliquot returned to the centrifuge tube. The stirred solution was then brough to 50 °C using an oil bath and stirred for ~12 h. The analysis procedure was again repeated. The 48 wt% aqueous hydrofluoric acid (HF) solution was analyzed directly by transferring an aliquot of the solution via plastic pipette to a Teflon NMR tube liner

Procedure for preparation of H_2SiF_6 and aluminum solutions

To each of four 15 mL plastic centrifuge tubes containing a Teflon coated magnetic stir bar was added 5 mL of 35 wt% aqueous hexafluorosilicic acid (H_2SiF_6). In one tube each was added ~100 mg of either 30 μm aluminum powder, Ti_3AlC_2 MAX phase, aluminum trifluoride (AlF_3), or alumina (Al_2O_3). Each of the solutions was stirred at room temperature for 1 h. An aliquot of each solution was transferred via plastic pipette to a separate Teflon NMR tube liner for analysis by ^{19}F and ^{27}Al NMR spectroscopy.

Procedure for preparation of HF and aluminum solutions

To each of four 15 mL plastic centrifuge tubes containing a Teflon coated magnetic stir bar was added 5 mL of 48 wt% aqueous hydrofluoric acid (HF). In one tube each was added ~100 mg of either 30 μm aluminum powder, Ti_3AlC_2 MAX phase, aluminum trifluoride (AlF_3), or alumina (Al_2O_3). Each of the solutions was stirred at room temperature for 1 h. An aliquot of each solution was transferred via plastic pipette to a separate Teflon NMR tube liner for analysis by ^{19}F and ^{27}Al NMR spectroscopy.

Procedure for preparation of Na_3AlF_6, KF, KHF_2, and HF control solutions in H_2SiF_6

Na_3AlF_6 solution in H_2SiF_6

In a 15 mL plastic centrifuge tube containing a Teflon coated magnetic stir bar was added 2 mL of 35 wt% aqueous hexafluorosilicic acid (H_2SiF_6). A 0.5 M solution of cryolite was prepared by dissolving Na_3AlF_6 (cryolite) (1 mmol, 0.2099 g) in the aqueous hexafluorosilicic acid with stirring for 10 minutes. An aliquot of the solution was transferred via plastic pipette to a Teflon NMR tube liner for analysis by ^{19}F and ^{27}Al NMR spectroscopy.

KF and KHF_2 solutions in H_2SiF_6

In two separate 15 mL plastic centrifuge tube containing a Teflon coated magnetic stir bars was added a saturated aqueous solution of either KF or KHF_2. Then 1 mL of 35 wt% aqueous hexafluorosilicic acid (H_2SiF_6) was added to each solution and stirred for 10 minutes. An aliquot of each solution was transferred via plastic pipette to a separate Teflon NMR tube liner for analysis by ^{19}F NMR spectroscopy.

HF solution in H_2SiF_6

A solution of HF and H_2SiF_6 was prepared by mixing equal volumes (1.5 mL) of concentrated 48 wt% aqueous hydrofluoric acid (HF) solution with 35 wt% aqueous hexafluorosilicic acid (H_2SiF_6) in a 15 mL plastic centrifuge tube containing a Teflon coated magnetic stir bar. The solution was stirred for 10 minutes. An aliquot of the solution was transferred via plastic pipette to a Teflon NMR tube liner for analysis by ^{19}F NMR spectroscopy.

^{27}Al and ^{19}F NMR spectroscopic analysis of Al and/or F containing controls (Figure S27) was used to further substantiate the prospect that H_3AlF_6 is the primary byproduct of the H_2SiF_6 etching process. This was undertaken by comparing the ^{27}Al and ^{19}F NMR spectra of the reaction of Ti_3AlC_2 MAX with H_2SiF_6, Figure S27 (a) row 2 and Figure S27 (b) row 2 respectively, with the ^{27}Al and ^{19}F NMR spectra of a solution of Na_3AlF_6 (cryolite) in H_2SiF_6, Figure S27 (a) row 3 and Figure S27 (b) row 3 respectively, as both an Al and F control. The ^{27}Al NMR spectra of Ti_3AlC_2 MAX or Na_3AlF_6 in H_2SiF_6 each displayed the same two overlapping peaks centered near δ 0.55 ppm to 0.92 ppm. The ^{19}F NMR spectra of both samples also displayed a nearly identical set of peaks at ~ δ -155 ppm and -156 ppm (Figure S27 (d)). Baseline ^{19}F NMR control, aqueous H_2SiF_6, shown again in Figure S27 (b) row 1, displays a strong peak at δ -130.09 ppm which is also seen in both sample solutions. The parity of peaks observed in the ^{27}Al and ^{19}F NMR spectra of the reaction of Ti_3AlC_2 MAX with H_2SiF_6 and a known AlF_6^{3-} complex support the assignment of H_3AlF_6 as the etching byproduct.

Further evidence that HF is not present in the H_2SiF_6 etching protocol in quantities detectable by ^{19}F NMR was obtained by analyzing fluorine controls proposed to produce HF in other reported Ti_3AlC_2 MAX phase etching procedures. Accordingly, solutions of HF, KF, and KHF_2 in H_2SiF_6 were prepared and examined by ^{19}F NMR (Figure S27 (b) rows 5-7). In each case, a new signal was detected in the region of ~ δ -161.6 ppm – δ -165.7 ppm, very near the baseline ^{19}F NMR chemical shift value for neat HF, δ -166.02 ppm (Figure S27 (b) row 4). The free fluoride ions available in HF, KF, and KHF_2 give rise, under acidic conditions, to a ^{19}F NMR signal attributable to HF. This same signal is not seen in any of the reactions of H_2SiF_6, shown in Figure 4 in the main text, and supports the proposed absence of fluoride ions or HF from the presented Ti_3AlC_2 MAX phase etching protocol using H_2SiF_6.

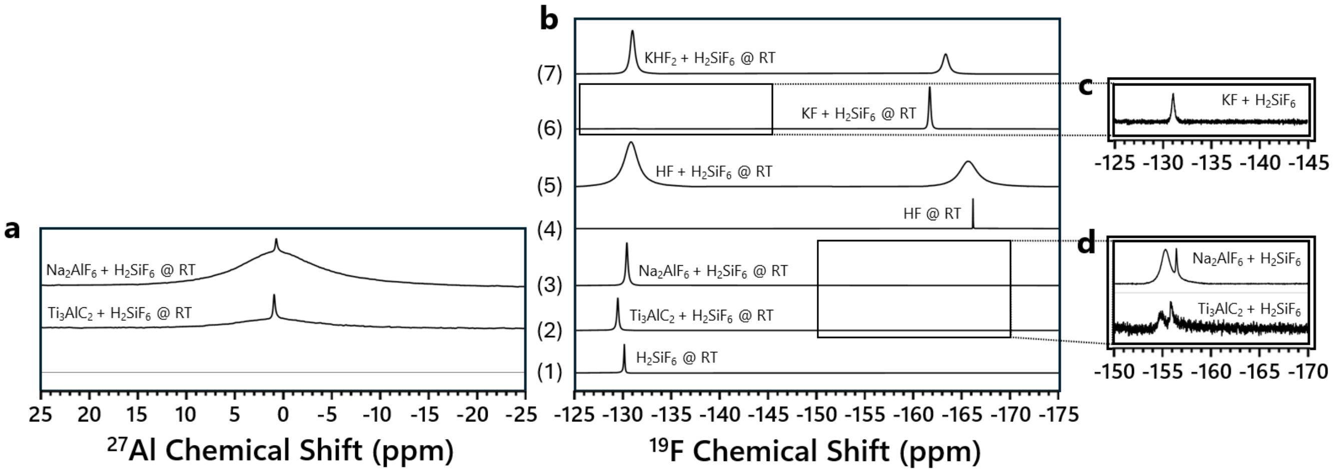

Figure S30: ^{27}Al and ^{19}F NMR spectroscopy study of aluminum and fluorine-containing controls. (a) ^{27}Al NMR spectra and (b) ^{19}F NMR spectra obtained from analysis of the aluminum and fluorine controls in H_2SiF_6 solution. (c) Highlighted portions of the ^{19}F NMR spectra demonstrating the similarity of the H_2SiF_6 etching byproduct and the Na_3AlF_6 (cryolite) control and (d) highlighted portion of the ^{19}F NMR spectra revealing the nearly complete disappearance of H_2SiF_6 from the reaction of KF with the H_2SiF_6 solution.

^{19}F NMR study on the effect of Al concentration in HF solution

Procedure for HF/Al concentration study

In a 15 mL plastic centrifuge tube containing a Teflon coated magnetic stir bar was added 2 mL of 48 wt% aqueous hydrofluoric acid. An aliquot of the aqueous HF was transferred via plastic pipette to a Teflon NMR tube liner for initial analysis. After analysis by ^{19}F NMR spectroscopy, the aliquot was returned to the plastic centrifuge tube. Aluminum foil was weighed (14 mg) and dissolved with stirring in the aqueous HF. An aliquot of the solution was transferred via plastic pipette to a Teflon NMR tube liner for initial analysis. After analysis by ^{19}F NMR spectroscopy, the aliquot was returned to the plastic centrifuge tube. This process was repeated following further additions of 13 mg and 27 mg of aluminum foil.

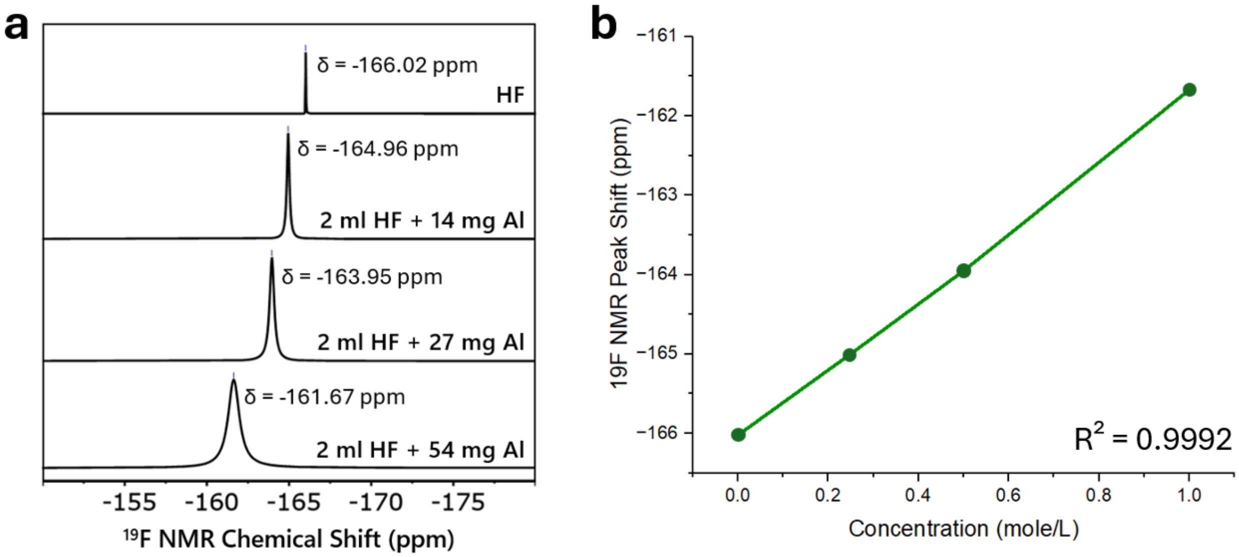

Figure S31. Study on the effect of aluminum concentration in aqueous HF solution. (a) ^{19}F NMR spectra from concentration study showing the downfield shift of the 19F NMR peak with increase aluminum concentration, with the neat HF standard shown for comparison. (b) Plot showing the correlation of ^{19}F NMR chemical shift with the concentration of aluminum in each tested solution.

Comparison of XRD patterns from H_2SiF_6 etching byproducts with standards

Procedure for isolation of H_2SiF_6 etching byproducts

In a 50 mL plastic tube containing a Teflon coated magnetic stir bar was added 5 mL of 35 wt% hexafluorosilicic acid. Aluminum foil was weighed (100 mg) and dissolved with stirring in the aqueous hexafluorosilicic acid solution. The mixture was stirred until all aluminum was visibly dissolved. An aliquot of the solution was transferred via plastic pipette to a Teflon NMR tube liner for initial analysis. After analysis by ^{19}F and ^{27}Al NMR spectroscopy, the aliquot was returned to the plastic centrifuge tube. After NMR analysis the reaction mixture was quenched using a saturated sodium carbonate solution which upon addition produced vigorous bubbling. After further addition of carbonate produced no further bubbling (~15 mL) the addition was stopped, and an aliquot of the solution was transferred via plastic pipette to a Teflon NMR tube liner for ^{19}F and ^{27}Al NMR spectroscopic analysis. The aliquot was returned to the plastic centrifuge tube, and the reaction mixture was dried and analyzed using XRD.

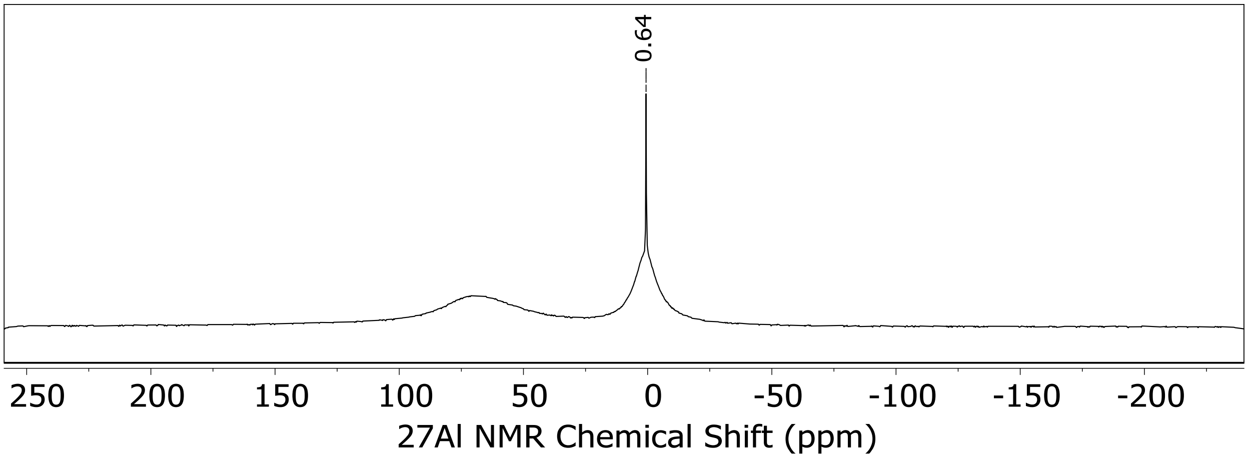

Figure S32. ^{27}Al NMR spectra of Al/H_2SiF_6 reaction prior to Na_2CO_3 quench

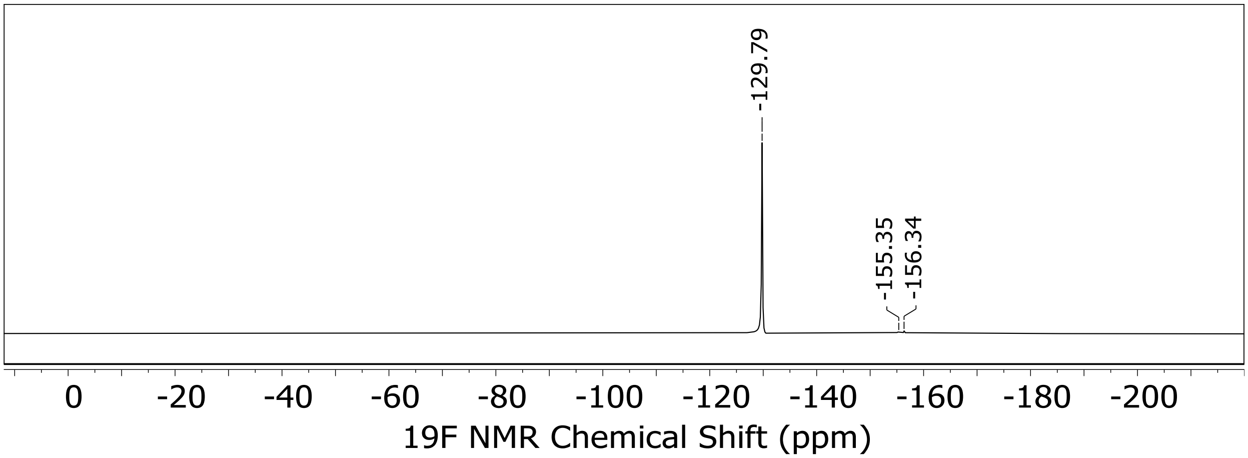

Figure S33. ^{19}F NMR spectra of Al/H_2SiF_6 reaction prior to Na_2CO_3 quench

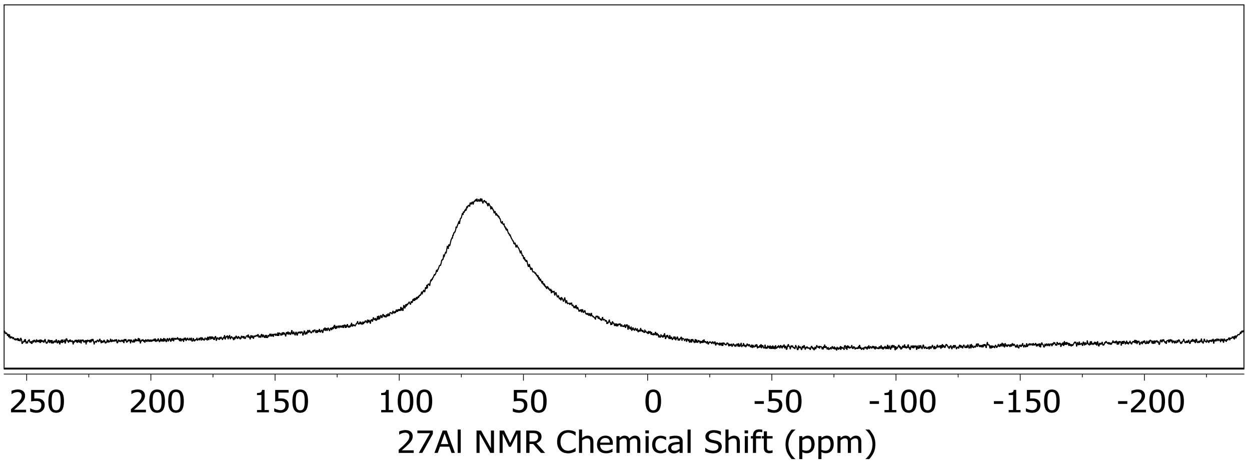

Figure S34. ^{27}Al NMR spectra of Al/H_2SiF_6 reaction following Na_2CO_3 quench

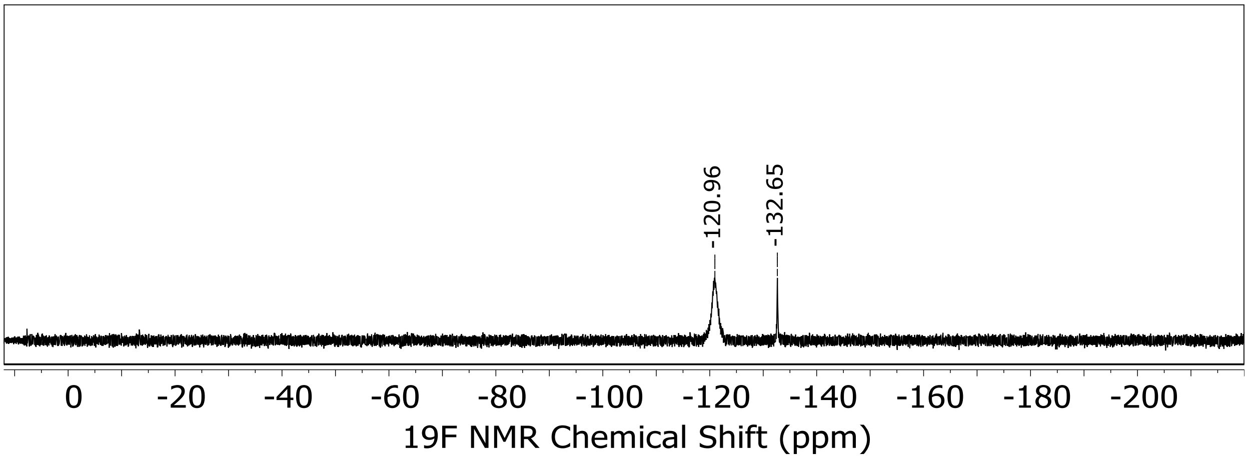

Figure S35. ^{19}F NMR spectra of Al/H_2SiF_6 reaction following Na_2CO_3 quench.

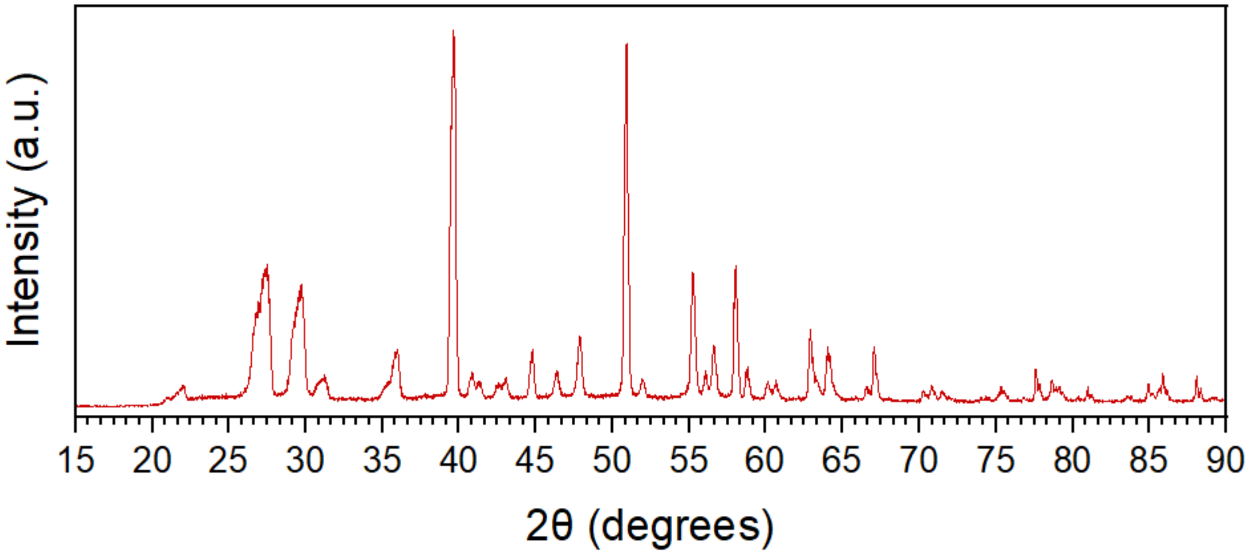

Figure S36. XRD pattern of commercial sodium hexafluorosilicate standard.

Table S5. Table of compounds identified in XRD pattern of commercial sodium hexafluorosilicate standard.

Entry	Compound	Formula	Percentage (%)
1	Sodium silicon fluoride	Na_2SiF_6	100

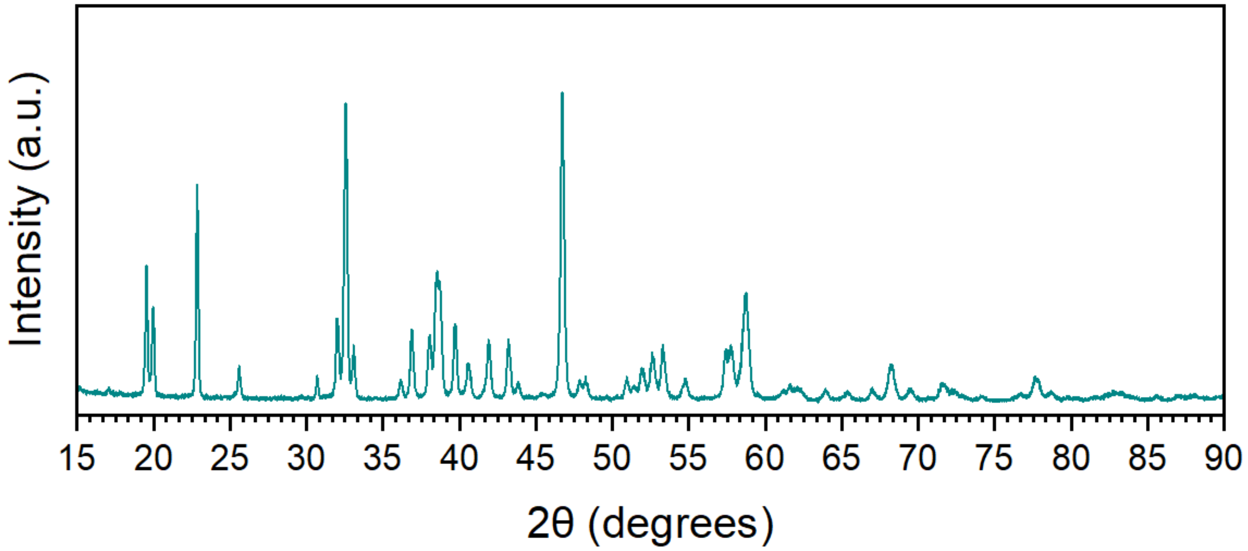

Figure S37. XRD pattern of commercial sodium hexafluoroaluminate (cryolite) standard.

Table S6. Table of compounds identified in XRD pattern of commercial sodium hexafluoroaluminate (cryolite) standard.

Entry	Compound	Formula	Percentage (%)
1	Sodium aluminum fluoride	Na_3AlF_6	82.4
2	Sodium aluminum fluoride	$Na_5Al_3F_{14}$	17.6

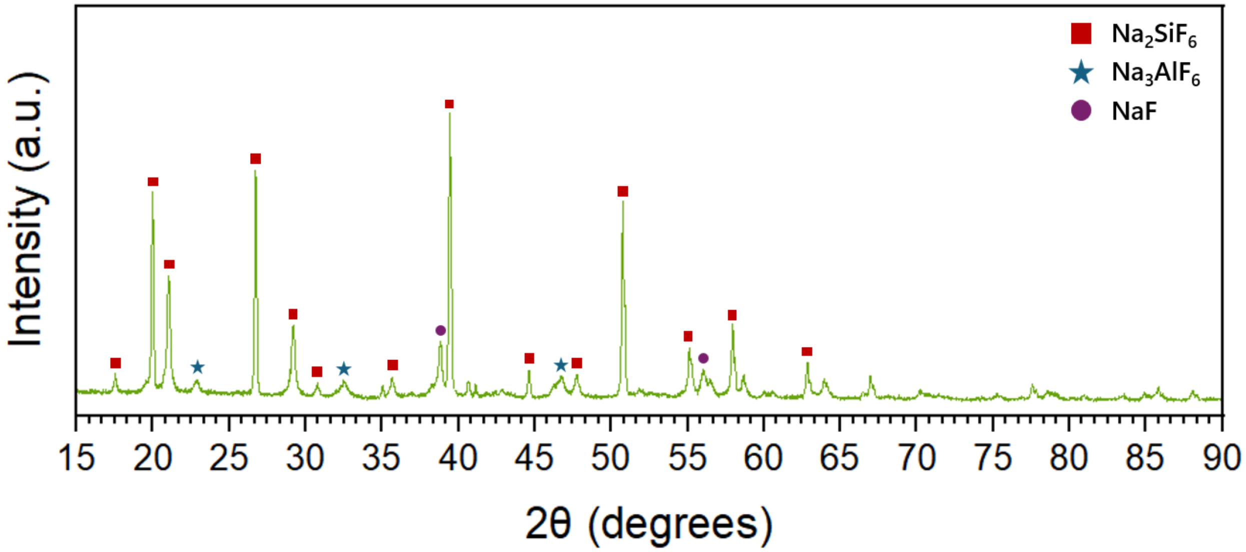

Figure S38. XRD pattern of Na_2CO_3 quenched reaction of hexafluorosilicic acid and aluminum.

Table S7. Table of compounds identified in XRD pattern of Na_2CO_3 quenched reaction of hexafluorosilicic acid and aluminum.

Entry	Compound	Formula	Percentage (%)
1	Sodium silicon fluoride	Na_2SiF_6	79.4
2	Sodium aluminum fluoride	Na_3AlF_6	11.4
3	Sodium fluoride	NaF	9.3

Preparation of calibration curve and etching reaction monitoring by ^{19}F NMR

Procedure for preparation of calibration curve

In a 50 mL plastic tube containing a Teflon coated magnetic stir bar was added 10 mL of 35 wt% hexafluorosilicic acid. Trifluoroacetic acid (0.15 mL, 1.9602 mmol) was added as an internal standard, and the mixture was briefly stirred. Then Na_3AlF_6 was weighed and added in increments, total mass and corresponding number of moles at each calibration point is given in Table S8 below. The solution was stirred until the Na_3AlF_6 added at a given increment was dissolved. An aliquot of the solution was transferred via plastic pipette to a Teflon NMR tube liner for analysis by ^{19}F NMR spectroscopy, with the aliquot transferred back to the 50 mL plastic tube following each measurement. The ratio of the integration of peaks areas of AlF_6^{3-} to trifluoroacetic acid are given in Table S8 below.

Table S8. Table of masses and mole quantities of Na_3AlF_6 contained in each calibration sample, and the corresponding ratio of AlF_6^{3-} to trifluoracetic acid peak integral areas determined for each sample using ^{19}F NMR spectroscopy.

Calibration point	Na_3AlF_6 mass (g)	Na_3AlF_6 moles (mmol)	Ratio of AlF_6^{3-}/TFA peak integrals
1	0.0225	0.1072	0.0268
2	0.0547	0.2606	0.0693
3	0.1114	0.5307	0.1688
4	0.1690	0.8052	0.2367
5	0.2176	1.0367	0.3098
6	0.2674	1.2739	0.3860
7	0.3254	1.5503	0.4785

Procedure for monitoring etching reaction

In a 50 mL plastic centrifuge tube containing a Teflon-coated magnetic stir bar, 35 wt% aqueous hexafluorosilicic acid (10 mL) was added. Trifluoroacetic acid (0.15 mL, 1.9602 mmol) was added as an internal standard, and the mixture was briefly stirred. Ti_3AlC_2 MAX phase was weighed (0.0250 g) and added with stirring to the aqueous hexafluorosilicic acid solution containing trifluoroacetic acid. The centrifuge tube was lightly capped, and the stirred suspension was brought to 35 °C using an oil bath. An aliquot of the solution was transferred via plastic pipette to a Teflon NMR tube liner for analysis by ^{19}F NMR spectroscopy, with the aliquot transferred back to the 50 mL plastic tube following each measurement. The time points examined, the ratio of the integration of peaks areas of AlF_6^{3-} to trifluoroacetic acid at each time point, and the corresponding quantity of AlF_6^{3-} (calculated using the linear regression line determined from the calibration curve ($y = 0.308118x – 0.00483266$, where y is the integral ratio and x is the resulting AlF_6^{3-} quantity)) are given in Table S9 below.

Table S9. Etching monitoring reaction timepoints examines, ratio of AlF_6^{3-} to trifluoracetic acid peak integral areas determined for each sample using ^{19}F NMR spectroscopy and each timepoint, and the corresponding quantity of AlF_6^{3-} contained at each time point.

Timepoint (h)	Ratio of AlF_6^{3-}/TFA peak integrals	Na_3AlF_6 moles (mmol)
4	0.1010	0.0263
8	0.1312	0.0356
20	0.2640	0.0765
28	0.2851	0.0830
44	0.5528	0.1655
68	0.9520	0.2885
92	0.8897	0.2693
96	0.9072	0.2747

References

41. Thakur, A.; Chandran B.S., N.; Davidson, K.; Bedford, A.; Fang, H.; Im, Y.; Kanduri, V.; Wyatt, B. C.; Nemani, S. K.; Poliukhova, V.; Kumar, R.; Fakhraai, Z.; Anasori, B., Step-by-Step Guide for Synthesis and Delamination of $Ti_3C_2T_x$ MXene. *Small Methods* **2023,** *7* (8), 2300030.
